# Unsupervised Clustering of Subcellular Protein Expression Patterns in High-Throughput Microscopy Images Reveals Protein Complexes and Functional Relationships between Proteins

**DOI:** 10.1371/journal.pcbi.1003085

**Published:** 2013-06-13

**Authors:** Louis-François Handfield, Yolanda T. Chong, Jibril Simmons, Brenda J. Andrews, Alan M. Moses

**Affiliations:** 1 Department of Computer Science, University of Toronto, Ontario, Canada; 2 Department of Molecular Genetics, University of Toronto, Ontario, Canada; 3 Department of Cell & Systems Biology, University of Toronto, Ontario, Canada; Carnegie Mellon University, United States of America

## Abstract

Protein subcellular localization has been systematically characterized in budding yeast using fluorescently tagged proteins. Based on the fluorescence microscopy images, subcellular localization of many proteins can be classified automatically using supervised machine learning approaches that have been trained to recognize predefined image classes based on statistical features. Here, we present an unsupervised analysis of protein expression patterns in a set of high-resolution, high-throughput microscope images. Our analysis is based on 7 biologically interpretable features which are evaluated on automatically identified cells, and whose cell-stage dependency is captured by a continuous model for cell growth. We show that it is possible to identify most previously identified localization patterns in a cluster analysis based on these features and that similarities between the inferred expression patterns contain more information about protein function than can be explained by a previous manual categorization of subcellular localization. Furthermore, the inferred cell-stage associated to each fluorescence measurement allows us to visualize large groups of proteins entering the bud at specific stages of bud growth. These correspond to proteins localized to organelles, revealing that the organelles must be entering the bud in a stereotypical order. We also identify and organize a smaller group of proteins that show subtle differences in the way they move around the bud during growth. Our results suggest that biologically interpretable features based on explicit models of cell morphology will yield unprecedented power for pattern discovery in high-resolution, high-throughput microscopy images.

## Author Summary

The location of a particular protein in the cell is one of the most important pieces of information that cell biologists use to understand its function. Fluorescent tags are a powerful way to determine the location of a protein in living cells. Nearly a decade ago, a collection of  yeast strains was introduced, where in each strain a single protein was tagged with green fluorescent protein (GFP). Here, we show that by training a computer to accurately identify the buds of growing yeast cells, and then making simple fluorescence measurements in context of cell shape and cell stage, the computer could automatically discover most of the localization patterns (nucleus, cytoplasm, mitochondria, etc.) without any prior knowledge of what the patterns might be. Because we made the same, simple measurements for each yeast cell, we could compare and visualize the patterns of fluorescence for the entire collection of strains. This allowed us to identify large groups of proteins moving around the cell in a coordinated fashion, and to identify new, complex patterns that had previously been difficult to describe.

## Introduction

High-content screening of fluorescently tagged proteins has been widely applied to systematically characterize subcellular localizations of proteins in a variety of settings [1]. Because they employ automated liquid handling and high-throughput microscopy, these experiments result in large numbers of digital images. Previous work has demonstrated that automated image analysis approaches based on machine-learning can classify these images into groups with shared subcellular localization patterns [2]. These approaches are typically ‘supervised’ in that they rely on predefined sets of example ‘training’ images for each pattern of localization to learn specific discriminative information that defines each class [3].

In contrast, unsupervised methods offer a more exploratory approach to high-throughput data analysis in which it is not necessary to predefine patterns of interest, and therefore can discover new patterns. This also enables the analysis of patterns that are very rarely observed, which typically are hard to capture in supervised analysis as a suitable training set for classification is difficult to construct [1]. Unsupervised analysis also has the advantage that it is unbiased by prior ‘expert’ knowledge, such as the arbitrary discretization of protein expression patterns into easily recognizable classes. For these reasons, unsupervised cluster analysis has become a vital tool of computational biology through its application to genome-wide mRNA expression measurements [4]–[7], and protein-protein interaction data [8]. It has also been applied in automated microscopy image analysis [9]–[13] where it has been shown to provide complementary capabilities to supervised approaches.

Here we apply unsupervised analysis to a set of high-resolution images of 4004 yeast strains, where each strain contains a different fluorescently tagged protein [14]. Because localization classes are not defined in advance, one difficulty is to identify a set of image features that reliably distinguish classes of protein expression [10]. Further, in order to allow identified statistical patterns to be directly related to our understanding of cell biology, we sought to define a small set of simple biologically interpretable measurements. This is in contrast to many automated image analysis approaches that use a large number of image features, which are typically used for object recognition in photographs [15], [16]. Although these features can be used to build powerful classifiers, the nature of the discriminative information does not need to be intelligible to allow class label recovery [3].

Recent work has demonstrated the power of incorporating cell-cycle stage into proteomics analysis (e.g., [12], [13], [17], [18]). Several studies have identified proteins whose abundance and localization change over the cell-cycle in mammalian cells. Furthermore, unsupervised analysis has been applied to identify novel, unexpected patterns. In general, these approaches have been applied to time lapse movies of mammalian cells, although it is also possible to acquire dynamic data from still images of mammalian cells [18].

One advantage of budding yeast as a model organism is that it shows stereotypical cell-cycle dependent morphological changes, which can be used to infer cell-stage based on cell morphology in still images of asynchronous cells. Previous work has demonstrated the feasibility of uncovering and analyzing yeast morphology using automated image analysis methods [19], [20]. Although the identification of cell boundaries in images has been shown to be unnecessary for subcellular localization classification [2], [21], [22], in order to extract dynamic protein expression profiles based on changes in cell morphology, in this work we sought to accurately identify individual cells. Here, we use an explicit model of yeast cell shape in order to (1) rapidly identify cells in high-resolution images, even when they occur in clumps, (2) obtain a probabilistic confidence measure for the identified cells and (3) define biologically interpretable measurements that describe protein expression in each cell over space and time.

We show that many previously defined subcellular localization patterns can be recognized in an unsupervised hierarchical cluster analysis. We find that protein complexes and small functional protein classes, which are not typically associated with their own subcellular localizations, cluster together in this analysis. Based on these observations, we show that the resolution of the hierarchical clustering is significantly higher than previous manual subcellular location assignments to discrete classes [14]. Further, we gain global insight into the cell stage dependence of protein localization; for example, we find a large cluster of nuclear proteins that seem to appear in the bud at a clearly defined time, which we believe corresponds to the inclusion of the nucleus in the daughter cell. Finally, we identify groups of proteins that show complex, dynamic patterns of localization that can not easily be predefined or described using simple localization classes; for example, many of the subunits of the exocyst complex are seen to localize to the bud periphery while the bud is small, but then move to the bud neck as the bud grows.

## Results

### Model-based identification of yeast cells

Starting with a collection of 4004 strains where each protein has been systematically tagged with green fluorescent protein (GFP) [14], a red-fluorescent protein (RFP) which appears everywhere in the cell was introduced into each strain using SGA [23]. These strains were then imaged in quadruplicate at high resolution to generate two-channel fluorescent images (see Methods). The RFP was introduced to facilitate automated analysis, as it provides both a signal for cell segmentation, as well as an internal control for methodological variation in fluorescence measurements.

#### A fast, accurate computational pipeline to identify yeast cells in high-resolution microscopy images

**Figure 1:**
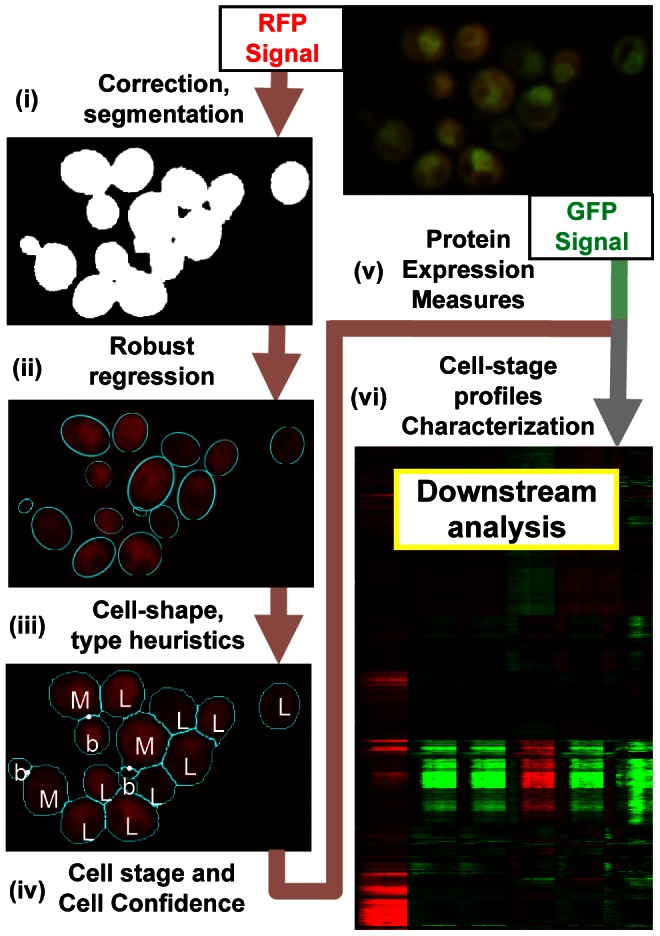
Pipeline of the methods used in this work. The identification of cells, assignment of cell type, cell stage and the estimation of cell confidence is based solely on the intensities of the RFP marker present in all strains. Please refer to the Results and Methods for descriptions of steps (i)–(vi). The cell type, stage and confidence are then used in conjunction with the GFP signal from tagged proteins in each strain in order to compute biologically interpretable features of protein expression.

One challenging aspect of automated microscopy image analysis is the presence of clumps of cells that makes the identification of individual cell boundaries difficult for conventional approaches, such as seeded watershed algorithm [16]. In our case, cell boundaries are inferred from the RFP alone, whose mean value varies from cell to cell and is often lower in vacuoles than in the spaces between cells, which implies that there is no RFP intensity level that systematically separates cells from each other. We therefore first segment the RFP image and obtain foreground regions that contain either single cells with no neighbours or clumps of cells (see Methods). In order to find the number of objects within each foreground clump, we use robust regression to fit ellipses to the clump (Figure 1, ‘Robust regression for ellipses’ in Methods). As it has been noted that combinations of segmentation methods are more powerful [24], we use the fitted ellipse coordinates to join fragments that are produced by the watershed transformation (see ‘Cell Shape’ in Methods). We compared the performance of our cell identification procedure to a manual assessment for a small fraction of the image collection and we find good agreement for ellipse size and center coordinates (see ‘Evaluation of cell identification performance’ in Methods).

**Figure 2:**
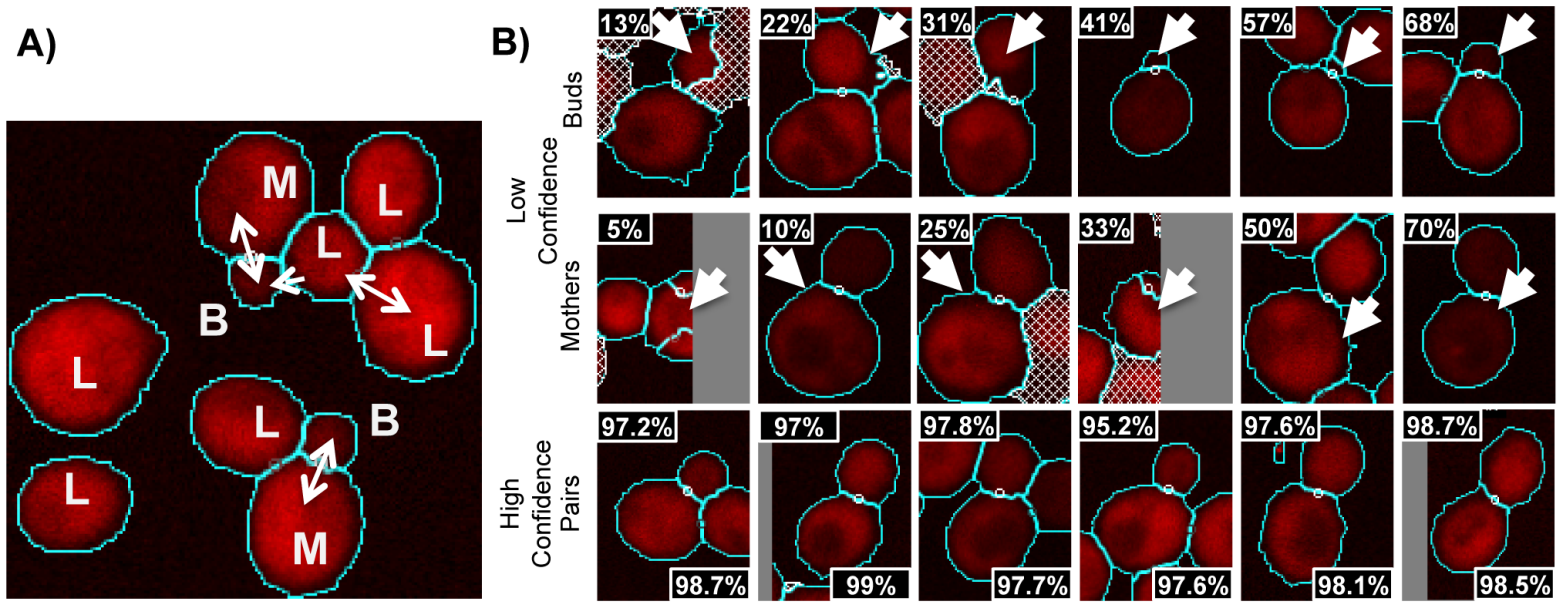
Yeast cell identification. a) Shows the mother-bud assignment heuristic. Pairs of circular objects that reciprocally have largest and smallest sizes among neighboring areas are said to be ‘mother’ cells (indicated by M) and ‘bud’ cells (indicated by B, mother-bud pairs indicated by bidirectional arrows), unless the potential ‘bud’ cell has a smaller neighbor than itself (indicated by a unidirectional arrow). Any other cells are labelled as ‘lone’ cells (L). b) Example of low and high confidence objects. The cyan lines in each image represent the cell contours produced, and the white dots indicate the predicted bud neck position. The dashed objects represent obvious artifacts that were filtered using thresholds (See text for details). Objects on the edge of images were not automatically filtered out, but are expected to have low confidence.

Since cells that are undergoing the budding process are better characterized by a pair of ellipses [25], we expect the above approach to identify bud and mother cells as separate objects. We therefore assigned a ‘type’ to each object: either artifact or one of three cell types (‘mother’, ‘bud’ or ‘lone’ cell). We first apply thresholds based on object size and shape to filter out the majority of obvious artifacts (see ‘Cell confidence’ in Methods). Then, the remaining objects were assigned types using a simple heuristic based on the cell sizes (Figure 2). Mother-bud pairs were defined as reciprocally smallest and largest adjacent cells, and in addition buds were not allowed to have any smaller neighboring object. Any other cell is considered unbudded or ‘lone’. With this definition, a mother-bud pair may be independent cells in G1 phase that are found to be adjacent: we still consider them as a pair since it is likely that such a connection existed in the very recent past if one of the two cells still small. In total, we characterized 405359 mother-bud pairs, and 494680 remaining lone cells, so that a total of 1.3 million cells were identified.

#### A confidence measure for automatically identified cells

Because automated identification of clumped cells in images with artifacts is a challenging computational task, we expect a fair fraction of the identified objects to be misidentified objects and/or non-trivial artifacts. Indeed, close examination of example images revealed a significant number of artifact classes: Noise in image corners, ruptured cells, cells that lost RFP, defective CCD pixels, contamination, and out of focus objects were sometimes erroneously identified by our pipeline. We therefore sought to develop a statistical measure to summarize our confidence that each identified object was really a yeast cell.

Instead of trying to characterize each artifact class, we defined 3 quality measures based on object shape and contour, which have known distributions for circular or ellipsoidal objects (see ‘Cell confidence’ in Methods). We also use the mean RFP signal within the object as an additional quality measure. We model variation in each quality measure using a Normal distribution whose parameters are a function of object size and infer parameters using a set of cell contours obtained from the set of manually fit ellipses (see Methods). A uniform distribution is used to model the quality measures from ‘non-cell’ objects, allowing us to compute the posterior probability that an object is a cell under the model that the objects in our images are drawn from a two-component mixture of cells and non-cells:
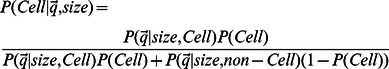
(1)where 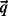 is the vector of quality measures and RFP intensity, and 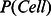 is a mixing parameter that can be thought of as the prior probability for an object to be a properly identified cell. We use EM to re-estimate that mixing parameter, while the cell class parameters are inferred from our set of manually identified cells and are not updated. We refer to this posterior probability as the ‘cell probability’ for each individual cell. The majority of cells in the images show high-confidence (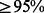) (Suppl. Figure S1). We define the probability of a mother or bud as the product of the two cell probabilities. We also allow these cells to be partially assigned to the lone cell class based on the cell probability of the putative related mother or bud. By analyzing a set of 139 manually identified artifacts, we found that filtering objects based on cell probability preferentially excludes artifacts (Suppl. Figure S2). However, we also found that small buds typically have lower cell probability (Suppl. Figure S1B), so defining cell confidence thresholds also preferentially filters small buds. Hence, we use these cell probabilities to weight individual cells when computing averages over cell populations.

### Quantitative characterization of cell cycle dependent protein localization

#### Describing protein expression using interpretable measurements

We next sought to characterize the protein expression phenotype using a small number of measurements that are biologically interpretable. The intensity of GFP signal in each cell relates to the level of protein expression [26], [27]. Therefore, as a first measurement, we use the ratio of total GFP intensity to RFP intensity within in each cell area.
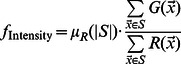
(2)where 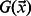 and 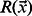 are the GFP and RFP intensities in the image at coordinate 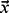. The 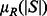 is the expected RFP intensity as a function of the cell area and ensures that the intensity ratios are comparable for cells of different sizes. This was necessary to correct for a systematic dependence of RFP intensity on cell size, which was characterized using the entire collection of identified cells (see ‘Protein expression measurements’ in Methods).

**Figure 3:**
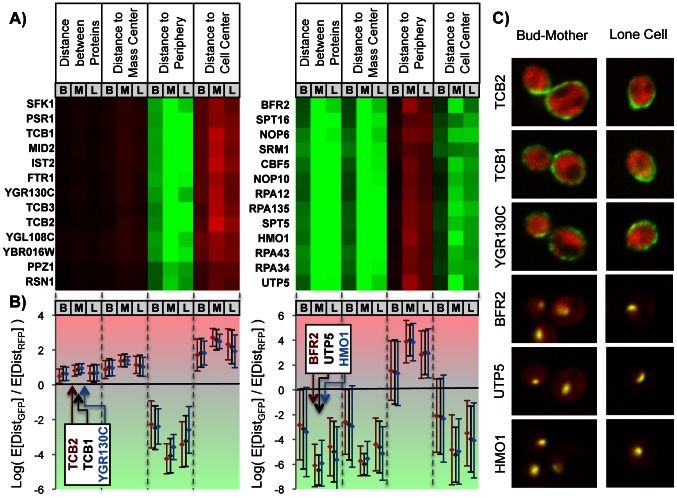
Morphological distances. a) Heatmap of the mean morphological distance features for each of the 3 cell classes automatically labelled: ‘bud’, ‘mother’ and ‘lone’ (columns indicated by ‘B’, ‘M’ and ‘L’ respectively). The proteins at the two extremes are enriched in cell periphery and nucleolus proteins. b) Three examples of the morphological distances extracted from the heatmap. Although the heatmap only shows the mean, we also compute the standard deviation (error bars). c) Examples of cells from the strains indicated in b). The spread of GFP fluorescence is greater than the RFP for the first three proteins, and less than RFP for the last three.

We define an additional set of 5 distance measures that characterize the spread of the protein within the cell (Figure 3). Assuming GFP intensities are proportional to protein amount, we can define the probability that a randomly chosen protein is located at a certain pixel coordinate as the fraction of protein found in that pixel. We compute this at each coordinate 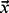 as the ratio of pixel intensity, 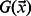, to the sum of the pixel intensities for that particular cell 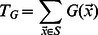, where ‘
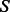
’ is the set of pixel coordinates that are within the area of a cell. Using this probability distribution over coordinates 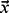, we derive the expected value for geometrical distances with respect to the position of a randomly selected protein. For example, for a pixel at coordinate 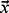, the distance to the cell center is given by 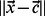. Therefore, we can define the expected distance of protein to the cell center:
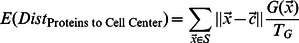
(3)Similarly, we define the average distance between proteins, to the protein mass center, to the cell center, to the cell periphery, and to the bud neck (see ‘Protein expression measurements’ in Methods). We refer to these measurements as expected distances, but it is important to note that they are actually estimates of protein proximities in 2-dimensional images and do not necessarily reflect the true 3-dimensional proximities. Nevertheless, these distances are easily interpretable summaries of protein expression patterns. In order to compare these expected distances between objects of different sizes, we also compute the distances for the RFP signal in each object and use these to normalize the distances obtained for the GFP signal (eq. 4). We report the log ratio of the expected distances, so that a negative value implies that distances are smaller for the GFP-tagged protein than the approximately uniformly expressed RFP and a positive value indicates that distances are greater for the RFP than for the GFP-tagged protein. While distance log ratios are dimensionless quantities, we refer to these 5 ratios as ‘morphological distances’ to emphasize that they measure the spatial spread in GFP intensity within each cell. For example, the ‘morphological distance’ to the bud neck, 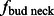 is defined as:
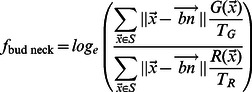
(4)where 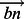 is the coordinates of the bud neck, 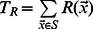 and 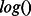 is the natural logarithm.

To analyze and display the morphological distances extracted for each cell for each GFP-tagged strain, we averaged the log ratios over the cells of each type (weighting cells by their cell probabilities) and display these averages as a heat map (e.g., Figure 3). In these heatmaps, red indicates positive values (i.e., on average greater values for the GFP-tagged protein than for the RFP) and green indicates negative values (i.e., on average smaller values for the GFP-tagged protein than for the RFP).

To illustrate the use of our morphological distances, we clustered the GFP-tagged strains using averages of the 4 distances (see ‘Protein expression measurements’ in Methods) for each of the 3 types of cells. As expected, clusters of proteins that show the smallest relative distance (i.e., closest) to the cell center were previously reported to be localized to the nucleolus and, on the other hand, the proteins displaying a large relative distance to cell center were previously reported to localize to the cell periphery (Figure 3). In contrast, if we consider the distance to the cell periphery, we see the opposite pattern, where nucleolar proteins show maximum distances, and cell-periphery proteins show minimum distances. This illustrates that the values we obtain for these expected distance features are related in a relatively simple way to spatial expression pattern of the protein. We note that this result does not imply that the morphological distances are superior to previously defined image features [2], [28] with respect to classifying subcellular locations; in fact, simple classifiers based on the morphological distances are less accurate (data not shown).

#### Automatic assignments of cell stage based on bud size

In addition to the cell type label, we consider the size of bud objects as a cell stage indicator for both the bud object and its corresponding mother cell. To infer ‘time series’ from our still images, we use the estimated area of each bud as a numerical represention over a continuous range of cell stages. In order to define a common basis for comparison of protein expression, we then use local regression (LOESS [29]) to estimate the mean and variance of feature measurements for mother-bud pairs at 10 selected ‘time’ points (see ‘Time profiles’ in Methods). The previously defined cell probability is used to weight each datapoint in these ‘time series’. (Eq. 21). To test the reliability of these ‘time series’ estimates, we performed a leave-one out jackknife resampling [30]. We found that the robustness of the ‘time series’ depends strongly on the number of identified mother-bud pairs, as expected. For the vast majority of proteins (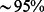 of proteins), the sampling variance estimated from the jackknife rarely (3.9% of the time) accounts for more than 2% of the variance observed in the estimate at any time point (Suppl. Figure S3A). In contrast, for a small fraction (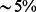) of proteins for which we have less than 26 mother-bud pairs identified, the weighted ‘time series’ estimates are much less reliable. We note that the use of confidence measure as a weight, as opposed to a threshold that filters undesirable data, produces ‘time series’ that have lower sampling variance (Suppl. Figure S3B).

**Figure 4:**
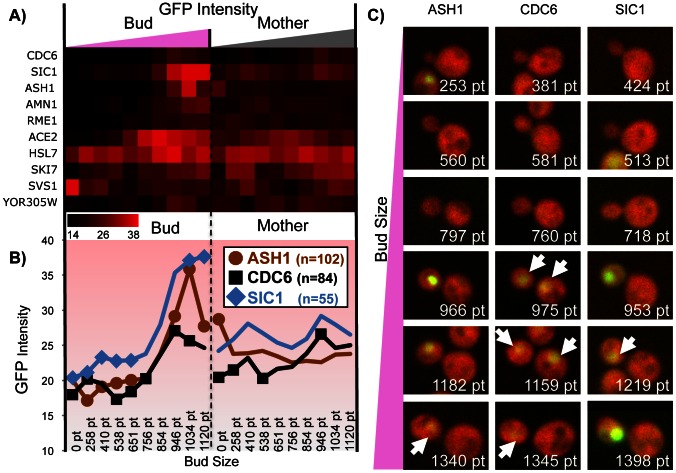
Intensity and time profiles. a) GFP intensity heatmap for several protein whose abundances are known to be cell-cycle dependent. b) Profiles for 3 proteins showing significantly higher expression level in large buds. ‘n’ is the number of mother-bud pairs used to infer each time series. 26 out of the 60 time points (indicated with markers) show coherent cell-stage specific deviation (permutation test, See Suppl. Figure S4). c) Examples of mother-bud pairs with the computed pixel size (pt) of the bud object (identical RFP/GFP intensity scale). The displayed cells were manually selected and then ordered by the computed bud size. Arrows indicate nuclear localization at lower intensity.

To test whether our estimates of cell stage based on bud size were reporting useful information, we examined the GFP intensity ‘time series’ (estimated as described above) for proteins whose quantity is known to vary over the cell cycle (Figure 4). For example, Cdc6 [31], Sic1 [32] and Ash1 [33] have been reported to be targeted for degradation by the SCF, a ubiquitin ligase that degrades target proteins at the G1/S transition [34]. Remarkably, these three proteins show similar variation in their intensity profiles, supporting the idea that our estimates of GFP as a function of cell stage are reflecting underlying biological variation in protein abundance. To test the statistical significance of these observations, we randomly permuted the cell stage estimates and recomputed the ‘time series’. We found that the coherent variation in the ‘time series’ estimated from the real data far exceeds what is typically observed in the permutations (Suppl. Figure S4). For example, for Cdc6, of the 6 of 10 points in the bud ‘time series’ and 4 of the 10 points in the mother ‘time series’ fall within the 5% tail of the distribution observed in the permutations (compared to 1 expected to fall in the 5% tail by chance). In all, for these three proteins 26 of 60 time points fall in the 5% tail (compared to 3 expected by chance). This shows that for these proteins whose levels are known to vary over the cell cycle, the variation observed in the ‘time series’ is statistically significant.

**Figure 5:**
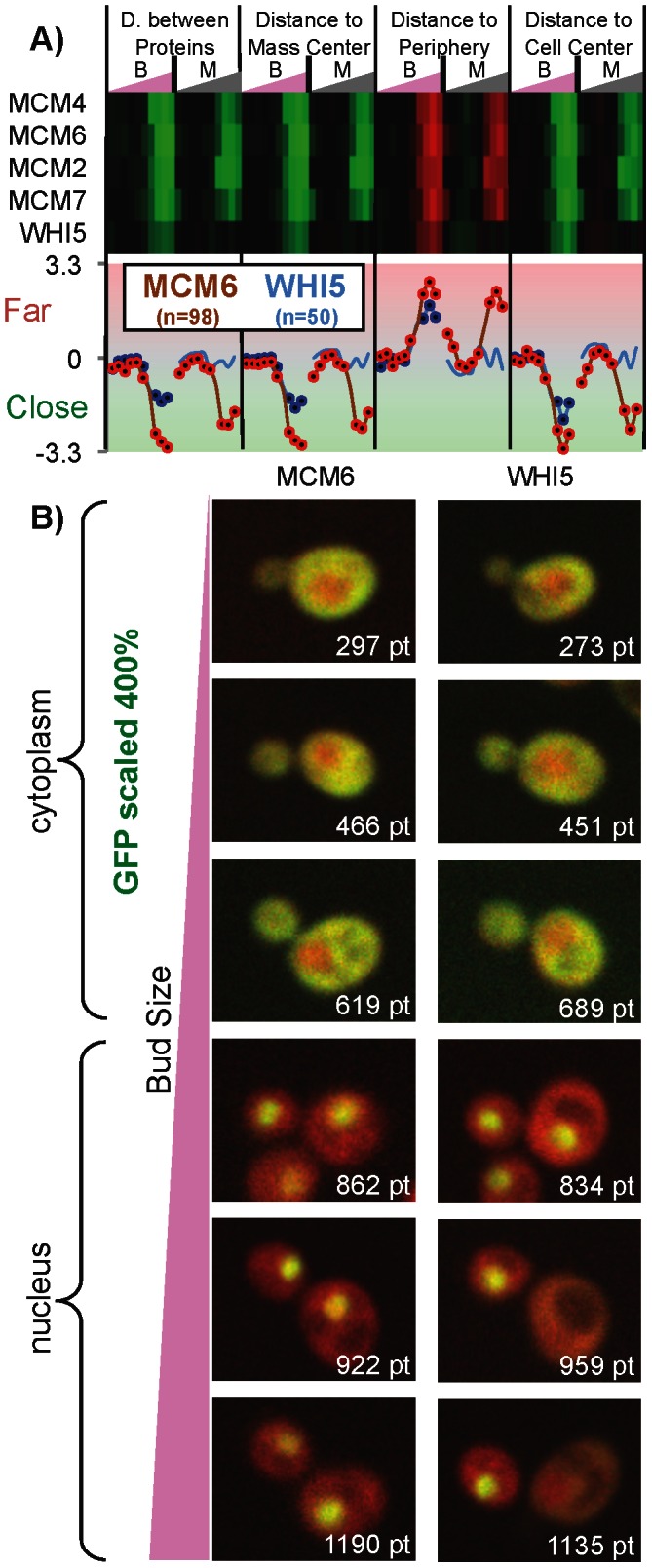
Time profiles of morphological distances. a) Top panel shows a heatmap of the morphological distances in bud and mother cells indicated as B and M, respectively. Bottom panel shows the data for two of these proteins as line graphs. The reported morphological distances are variance normalized. MCM complex subunits and Whi5 display a cell-cycle dependent subcellular location; cytoplasmic for small buds, nuclear for large buds. ‘n’ is the number of mother-bud pairs used to infer each time series. Out of the 80 timepoints for each protein, 34 for Whi5 (blue traces), and 72 for Mcm6 (red traces) show significant cell cycle variation (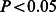, indicated as dark dots). b) Examples of mother-bud pairs that were ordered by the computed bud size (pt). The GFP channel was scaled between images to more clearly illustrate the change in subcellular location.

We estimated ‘time series’ for each of our 5 morphological distances and GFP intensity as described above for all of the bud and mother cell pairs. For each protein, we concatenate the 6 pairs of ‘time series’ into a ‘time profile’, which is a vector of 120 values. An example of a striking cell-cycle pattern is the profile observed for the subunits of the MCM complex (Figure 5), which is known to be exported from the nucleus at a particular cell stage by the activity of Clb/Cdc28 kinases [35]. This exclusion from the nucleus is captured by the distance features, since the protein gets closer to the cell periphery and, on the other hand, the average distance between proteins and to the to cell centre increases. This exclusion is observed in the mothers of small buds, so we can determine the size of the bud corresponding to the G2 to M transition, when the MCM complex nuclear localization signals are no longer specifically inhibited by Cdc28 (see figure 5B). Encouragingly, all 4 available members of this complex show this pattern (2 are missing from the GFP collection). This indicates that proteins displaying similar cell stage variation can be identified from their time profiles, despite the presence of noise in the images and heterogeneity in the distribution of identified cells on which the time profiles are based. Remarkably, we observe that another protein with a similar stage-dependent morphological distance profile is also known to have its localization is modulated by Cdc28 (Whi5 [36], see figure 5). Upon examination of the images, we observe a very similar expression pattern in bud cells for Whi5 and the MCM subunits, but that (in contrast to the MCM subunits) Whi5 nuclear localization is only rarely found in mother cells (Figure 5). This demonstrates the capacity of the generated profiles to capture cell-cycle dependence of changes in localization. Furthermore, that these proteins are all substrates of Cdc28 suggests that similarity in our profiles of morphological measurements may indicate common mechanisms that control subcellular localization, just as similar mRNA expression profiles are often used as evidence for common mechanisms of transcriptional control [4], [5].

#### Similarity between profiles of previously annotated localization classes reflect biological relationships

To get a global sense of whether the profiles in our biologically interpretable feature space reflect the biological similarity of protein expression patterns, we computed the average profiles for all the proteins within previously identified subcellular localization classes [14] (see ‘Class profiles’ in Methods). Because each profile represents a multivariate Normal distribution, where we estimate mean and standard deviation over the observed cells for 10 time points for each of the 6 features, for the mother and bud, we measure the similarity between the mean profiles for each localization class (‘class profile’) using the Bhattacharyya distance (Eq. 24). Consistent with their biological relationships, we observe that the class profiles representing nuclear proteins are much closer to nucleolar and nuclear periphery localized proteins (Bhattacharyya distance = 5.41,2.39) than to the class profiles for cytoplasmic or cell periphery localized proteins (Bhattacharyya distance = 34.20,21.16, Suppl. Table S1). Clustering of these class profiles placed several biologically related classes adjacent to each other in the hierarchy. For example, profiles for Golgi, Early Golgi and Late Golgi were clustered together (Suppl. Figure S5). To confirm this result, for each group of biologically related classes, we compared the average Bhattacharyya distances within the groups of related classes to the distances between the classes in each group all other classes. We found that the distances between biologically related classes were significantly smaller (6.14 vs. 15.44, 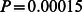, permutation test, Suppl. Figure S5). Taken together, these results show that distances in this interpretable feature space recapitulate the known biological relationships between localization classes.

### Unsupervised analysis of protein localization

**Figure 6:**
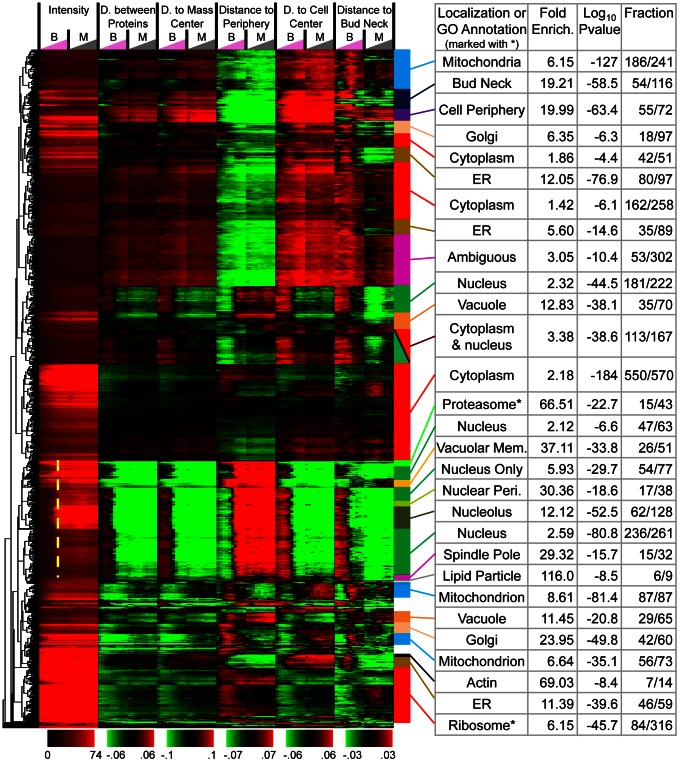
Time profile clustering result. A heatmap with 4004 GFP-tagged strains ordered using maximum likelihood agglomerative clustering based on the time profiles of protein abundance and 5 morphological measures. Within manually selected clusters (colored bars), the fraction of proteins in the cluster that have the same subcellular localization or GO Annotation (the latter indicated with stars) is listed under Fraction. Log p-values were computed using the hypergeometric distribution to test against the null hypothesis that the cluster was drawn randomly from the protein annotations. Fold enrichment indicates the ratio of the Fraction of proteins in the cluster with each annotation compared to that in the protein collection. Nuclear proteins appear in the bud at a specific time (dashed line).

Encouraged by the consistency and interpretability of our measurements relative to previous knowledge about yeast subcellular localization, we performed global unsupervised analysis of our time profiles of interpretable features for the mother and bud cells (Figure 6). To identify groups of proteins with similar patterns, we use agglomerative hierarchical clustering based on a maximum likelihood criterion [37] (see ‘Maximum likelihood agglomerative hierarchical clustering’ in Methods) because it does require the size (or number) of clusters to be specified, and we expect hierarchical relationships between functional classes, and a wide range in the number of proteins in each class. The hierarchical clustering results may be browsed online using the Java Treeview [38] applet at http://www.moseslab.csb.utoronto.ca/louis-f/unsupervised/.

#### Proteins in previously known localization classes cluster together

We performed a statistical enrichment analysis in order to compare our cluster analysis to previous knowledge about protein localization and function. We considered assignments of proteins to discrete localization classes from systematic manual assessments of the GFP collection [14] and GO annotations curated from the biological literature [39]. We found that many of our clusters were strongly enriched for GO annotations and previously identified subcellular localizations (Figure 6). We note that these results were not dependent on the clustering parameters or algorithm used, as similar results were obtained using other parameter sets or algorithms (Suppl. Table S2).

#### Nuclear, ER and mitochondrial proteins appear in the bud at specific cell stages

The unsupervised analysis of biologically interpretable features allows us to visualize a quantitative representation of protein localization over the cell cycle: we observe large clusters of proteins that appear in the bud sequentially. Most strikingly, in the clusters significantly enriched in nuclear proteins, protein expression is absent from the bud until approximately half-way through our time series (Figure 6). Other clusters also display cell-cycle dependent variation in all morphological distances, which appear to be specific to subcellular location. For example, the three mitochondrion enriched clusters show signal unusually far from the bud neck at the same time. Interpreting this pattern, we predict the presence of punctae in small buds, and inspection of the images confirmed this prediction (Suppl. Figure S6A).

**Figure 7:**
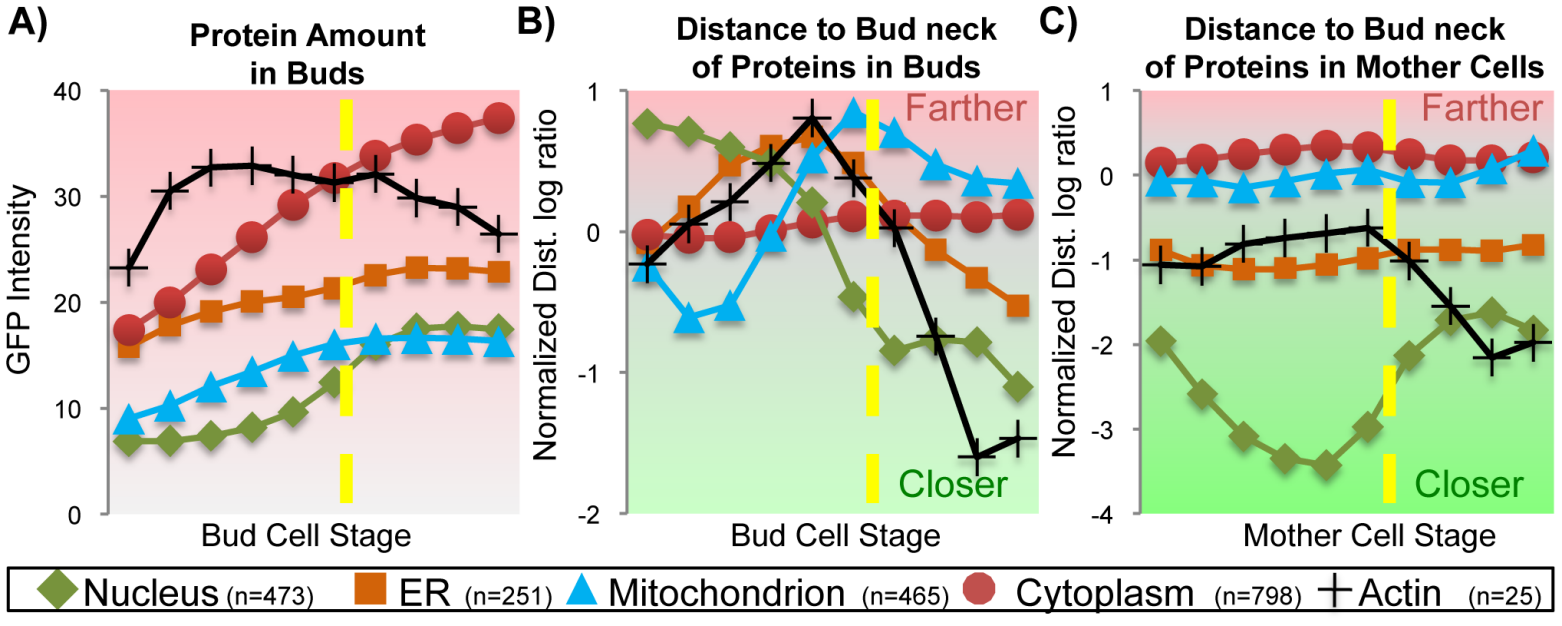
Subcellular location class profiles. a) Time series for protein abundance in buds. Nuclear proteins are the last to appear in the bud (dashed line). b) The spatial distribution of protein expression is highly variable in the growing bud cell. Organelles appear to be pushed from the bud neck at the time of the nucleus inclusion (dashed line). Note that the absence of nuclear protein in the bud leads to irrelevant variations in the morphological distance features, perhaps due to auto-fluorescence captured in the GFP channel. Actin proteins migrate from bud tip to bud neck (black traces). c) In the mother cell, organelles appear to maintain a typical distance to the bud neck, expect for the nucleus.

In order to confirm that the observed trends in the protein profiles are truly linked to the subcellular localization of the proteins, we used the class profiles (see ‘Class profiles’ in Methods) for each subcellular location (Figure 7). We observe that proteins from the nucleus, nuclear periphery and nucleolus are the last to appear in the bud. This is explained by the fact that DNA replication is occurring within the mother cell, and that the new nucleus has yet to be included in the bud. We note that in the bud cells, mitochondrial and ER proteins show elevated distances from the bud neck at the time of nucleus inclusion, and that a subset of the mitochondrial proteins are found close to the bud neck in the smallest bud objects (Figure 7b); this suggests that the mitochondria and ER may be included in the bud before the nucleus, and then pushed further from the bud neck as the nucleus occupies that position at the time of its entry into the bud. Interestingly, we also observe that the proteins of each organelle have typical distances in the mother cell to the current bud neck (Figure 7c). For example, the ER has been previously reported to stay close to the nucleus [40], and we observe that both the ER proteins are closer to the bud neck than the mitochondrial proteins (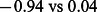, 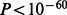, two-sample t-test) but not as close to the bud neck as the nuclear proteins (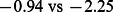, 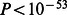, two-sample t-test).

We also observe the motion of the actin proteins in both the bud and mother cells, which agrees with previous observations: actin proteins localize at the bud periphery and then at the bud neck [14], [41]. Since the polarity of yeast cells is determined by the cell stage, and cell polarization is controlled via the action of the actin filaments [42], these results again indicate that our estimate of bud size is a good cell stage indicator, and that the order of biological events may be extracted directly from the class profiles. Although these patterns were discovered through interactive exploration of a particular clustering result, we note that these patterns correspond to very strong signals in the data and were also easily identified in clustering results derived from alternative similarity metrics or alternative usage of the confidence measure (Suppl. Figure S6B).

#### Proteins in functional classes and complexes cluster together

In our global analysis, we also observed clusters that were statistically enriched in annotations that do not correspond to subcellular localization classes or compartments (Figure 6). For example, translation is known to occur in the cytoplasm [43]. Nevertheless, we observe a cluster of 316 proteins where 86 (27%) correspond to structural components of the ribosome and a total of 121 (38%) are annotated as involved in translation. Consistent with the known cytoplasmic localization for the translational machinery, this cluster shows a similar overall pattern to cytoplasmic proteins, but can be distinguished because the average GFP intensity (presumably reflecting protein abundance) for these proteins is much higher than most other cytoplasmic proteins (Figure 6). As another example, we also noticed a cluster where 16 of 43 (37%) of proteins were subunits of the proteasome. This cluster also contains 6 of 14 (43%) proteins annotated as vacuolar ATP-ases. The pattern associated with this cluster shows high levels of protein abundance and is similar to that of nuclear proteins, but this is not sufficient to explain why these complex subunits are distinguishable from the remainder of the highly expressed nuclear proteins. The localization pattern for these proteins is more compact than other nuclear proteins, and we speculate that these complex subunits display similar, typical levels of compactness and this is captured in our morphological distances (Figure 6). These results suggest the possibility that a combination of a small number of interpretable features (e.g., cytoplasmic localization and high level of protein abundance) will define certain functional classes (see Discussion).

In order to report on the statistical significance of functional annotations in the hierarchical clusters, for each of the 2134 GO annotations that are shared by at least 2 proteins, we found the cluster within the hierarchy that has the most significant P-value. We used the sum the log of these P-values as a summary statistic, S, for the enrichment of annotations. For the real data we obtained S = −7078. To test whether this value was more extreme than what would be expected if the clusters were random, we permuted the genes while conserving the hierarchical topology 10000 times, and obtained S on average to be 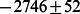 std. dev. Therefore, the observed value was 80 standard deviations away from the random expectation. Since we already have shown that the hierarchical clustering results contain clusters that are enriched in subcellular locations, this strong statistical significance is expected, as subcellular location and functional annotation of proteins are strongly connected. Therefore, we next tested whether functional annotations were enriched in our clusters beyond what could be explained from subcellular location enrichments alone. To do so, we again generated the distribution of S, but this time constrained the permutation so that proteins can be replaced in each iteration only if they share the same set of discrete subcellular location annotations [14]. Even with this constraint on the permutations, we obtain a 32.1 std. dev. lower value of S than in the permutations, and note that none of the 10000 permutations showed a more extreme value of S (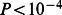).

To further demonstrate that the hierarchical clusters reflect functional information about the proteins (beyond what is contained in the discrete subcellular location annotations [14]), we repeated these permutation tests on subsets of the GO annotations partitioned based on the size of the set of proteins annotated according to that function in our list of proteins. Again, for both the constrained and unconstrained permutation tests, all sizes of GO annotation groups are found significant (we never observed such extreme values in the permutations, so all groups have 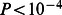, Suppl. Table S3). We also found similar results for Pfam domains and protein complexes, and extracted lists of protein groups that contribute to these two observations (Suppl. Tables S4, S5). To confirm that these results were not dependent on the particular clustering algorithm or parameter settings, we performed similar statistical analysis on clustering results obtained using different distance metrics or clustering methods and found similar results. (Suppl. Tables S3, S4, S5). These analyses imply that the biological information in the hierarchical clusters cannot be fully explained by the systematic subcellular location annotations [14], and, more importantly, that this unsupervised analysis must be capturing finer similarities in temporal and spatial expression for many groups of functionally related proteins.

These results do not imply that the unsupervised analysis allows prediction of subcellular location with accuracies on par with supervised methods. Nevertheless, these results show that there is more biological information in the subcellular localization patterns than is summarized by the previous annotations of localization classes. Therefore, we expect the unsupervised analyses to identify novel patterns that are biologically meaningful. We next sought to explore such novel patterns.

#### Dynamic distinctions between bud neck classes

**Figure 8:**
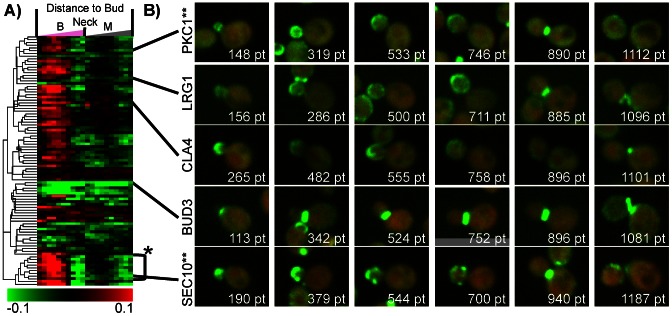
A cluster of 91 proteins displaying time profiles with variable distances to the bud neck. a) Heat map of the cluster displayed as in Figure 6. We observe several classes of dynamic patterns, which capture the localization to the bud neck and bud periphery. (*) 5 of the 8 subunits of the exocyst complex are found within 9 proteins. b) Examples of proteins with dynamic bud patterns. (**) The displayed GFP intensity was scaled down by 75%.

Because our analysis explicitly models cell stage, we can identify dynamic patterns where proteins move from one subcellular location to another. For example, we identified a cluster of proteins that showed a large range of distances to the bud neck, and for many of them, the distance to bud neck varied over the cell stage (Figure 8). In this cluster, we find a group of proteins that first appears in the periphery of the bud, and then migrates at a particular cell stage to the bud neck. Interestingly, these include Pkc1 and Lrg1 (Figure 8), which are both in the cell-wall integrity pathway [44]. Another functionally related group of proteins that shows the same dynamic pattern are the subunits of the exocyst complex (e.g., Sec10 Figure 8), but they appear to be more compact in small buds. This is in contrast to other profiles which represent proteins that always located at the bud neck. Unlike Pkc1, Lrg1 or the subunits of the exocyst, Bud3 shows a consistently small average distance to the bud neck (Figure 8). It can therefore be considered a pure ‘bud neck’ localization pattern, as opposed to Pkc1, Lrg1 and the exocyst subunits that are cycling from the bud periphery to the bud neck analogous to the way the MCM subunits cycle from the cytoplasm to the nucleus. This suggests that these dynamically changing bud-periphery to bud neck proteins have localization that is targeted by a shared cell-cycle regulatory mechanism. Yet another subtle variation on this theme is illustrated by proteins that are found specifically in the bud periphery, but do not migrate to the bud neck (e.g., Cla4, Figure 8). We speculate that these proteins lack a specific portion of the cell cycle regulation shown by Pkc1, Lrg1 and the exocyst subunits.

We also find in the same cluster 23 proteins that were not previously annotated in systematic studies as being bud-specific or actin [14], [16]. We predict that these proteins show dynamic patterns within the bud during its growth, and were difficult to describe using discrete annotations. For these proteins, SGD [45] annotations mostly disagree with previous systematic annotations (Suppl. Table S6). Among the 23 proteins, we find proteins that have functional links to other proteins known to be bud-specific, such as Ack1/YDL203C which is thought to function upstream of Pkc1 [46]. Looking at the images, Ack1 shows a pattern similar to Pkc1, with the difference that the protein abundance in the bud is not strong relative to the basal cytoplasmic expression in the mother cell (Suppl. Figure S7). Similarly, Msb3, Lte1 and Zds1 have been previously reported to show bud-related patterns in low-throughtput analyses ([47], [48], [49]) and are found in this cluster.

Hence, we hypothesize that proteins are found in this cluster (Suppl. Table S6) because they are showing various dynamic localization patterns with respect to the bud. Indeed by further inspecting the images we found a protein of unknown function, YDR239C, that shows a dynamic bud pattern similar to Ack1. This protein has not been previously characterized to localize to the bud periphery or bud neck and therefore represents a new positive prediction obtained from the unsupervised analysis. In contrast, visual inspection of other images reveals that some proteins in this cluster do not show obvious dynamic bud patterns. For example, Tpo3 was characterized as a cell periphery [14], [16] and plasma membrane [45] protein. The subcellular location of Tpo3 in our images is different than the dynamic bud patterns we previously described. Yet, it was clustered next to Rtk1, which appears in our images as a cell periphery protein that is is partially localized at the bud neck at the expected cell-stage (Suppl. Figure S7). This inclusion of Tpo3 was likely due to the similarity in the pattern of Tpo3 and Rtk1. This is expected of hierarchical cluster analysis, in that there are no hard delineations between the quantitative patterns (see Discussion).

This cluster illustrates pattern discovery using biologically interpretable features. We identified a group of proteins showing complex expression patterns that have been difficult to define previously. We believe this is due in part to the higher resolution of our images, as well as our ability to assign dynamic, quantitative patterns to these proteins. We note that not every protein in this cluster actually shows (as far as we can tell by inspecting the images) a dynamic bud pattern (Suppl. Figure S7). Nevertheless, we could relate the consensus pattern in this cluster (variation in our measurement of ‘average distance to bud neck’) to cell-cycle dependent migration from bud periphery to bud neck.

## Discussion

### In silico synchronization of yeast cells

Previous studies have demonstrated the feasibility of uncovering cell stage from images of unsynchronized cell populations, either from time lapse movies [17] or from still images [18]. We apply this approach to high-throughput still images of budding yeast. To do so, we devised a segmentation method to identify and separate the bud and mother cells, and uncover the cell stage based on measurement of the bud size. Our method depends critically on our estimates of bud size, and we show that the automatically estimated sizes were comparable to those obtained from manually identified cells. Several parts of the analysis may be improved. For example, since the bud-site selection is predetermined by the position of the preceding daughter cell [50], it could be used to help determine the correct mother-bud assignments. Similarly, a better model for the relation between daughter cell size and the cell cycle could be used to infer a more accurate estimate of cell stage.

### Probabilistic model yields confidence estimates

We presented a cell identification pipeline that includes a confidence measure which summarizes the probability that an object identified in our images is actually a correctly identified cell. To do so, we characterized the deviation of real cells from an elliptical model using several quality measures whose distribution for real cells we inferred from ellipses that had been manually fit to cells by eye. Our confidence measure allows us to distinguish correctly identified cells from artifacts and misidentified objects, without specifying what the nature of artifacts might be (Suppl. Figure S2). We believe that this type of approach for measuring the confidence of automatically identified objects in image analysis will be generally useful, because artifacts tend to vary between microscope, experiments and computational methods, whereas cell shapes are expected to be much more consistent. In addition, this confidence measure is explicitly defined as a posterior probability of an identified object to be a properly identified cell. This allows us to weight probabilistically data points according to the posterior probability. For classes of cells where our model does not fit as well, such as very early non-ellipsoidal buds, we expect to downweight all the data points, but we can still include information from these data points in our analysis. This is in contrast to the situation where we used a hard threshold to exclude artifacts. In that case, certain classes of cells are preferentially excluded (Suppl Figure 1B), and the statistical significance of downstream analyses is reduced (Suppl. Tables S2, S3, S4, S5).

### Quantitative descriptions of subcellular expression patterns

Typically, spatial patterns of protein expression are described by assigning labels [14] or functional annotations [51]. Such discrete classes are not sufficient to fully describe a protein's expression if it is present in quantitatively different localizations or abundances at different cell stages, or if a protein is simultaneously present in several locations with quantitatively different fractions [52]; because our approach assigns a quantitative expression profile to each protein, we can characterize protein expression at a finer scale than the resolution currently achieved by discrete classes. Approximating protein expression patterns as discrete classes has also led to challenges for computational analysis. For example, in previous work based on discrete classes [16], [21] many proteins are often filtered out because they have either been annotated as ‘ambiguous’ or are reported to be located in several localization classes.

Because we treat expression patterns quantitatively, our analysis identifies clusters of proteins that are significantly enriched in ‘ambiguous’ proteins and proteins that were manually annotated [14] as localized in multiple compartments. Furthermore, our analysis identified and organized a group of proteins that show complex patterns relating to the growth of the bud, that were not consistently annotated previously using discrete categorizations (Suppl. Table S6). To our knowledge no previous genome-scale analysis of still microscopy images has identified groups of proteins with subcellular localization patterns that change as a function of cell-stage, such as the MCM and exocyst complex subunits discussed above, although recent work on smaller collections of time-lapse images has demonstrated that functionally related proteins can be identified in unsupervised analysis of dynamic protein expression profiles [13].

### Clustering protein expression patterns

One limitation of cluster analysis is that the members of each cluster identified are not always consistent between different parameter settings, or different clustering methods. Indeed, the remarkably specific groupings corresponding to specific regulatory mechanisms (such as the clustering of all 4 of MCM complex subunits and of all 3 DNA replication factor A complex subunits) were not always observed when we varied the distance metric or clustering method used (Suppl. Table S4).

Despite these limitations, our analyses consistently identified clusters that were enriched in functional groups of proteins (Ribosome, Proteasome, DNA-damage pathway, exocyst complex, etc.; see Suppl. Table S4, S5) that are not usually associated with their own subcellular compartments. Because we used hierarchical clustering of interpretable features, we could see that these functional groups of proteins showed patterns of localization similar to those localized in the same compartment, but in each case showed subtle differences in pattern that allowed them to be distinguished. These results suggest that high-resolution images could be used directly for functional discovery as has been reported for mammalian cells [12].

This work demonstrates that accurately identifying large numbers of cells for each protein allows quantitative characterization of spatial and temporal characteristics of protein expression patterns and permits direct interpretation of image-based measurements without requiring human inspection of large numbers of images to train classifiers. Our analysis gives new insight into the relationship between protein function and protein expression patterns inferred from high resolution microscope images.

## Methods

### High-resolution yeast image dataset

Using yeast synthetic genetic array technology [23], a new GFP collection was generated from the existing collection [14]. In this new collection, a highly expressed RFP (a tdTomato [53] fluorescent protein from the constitutive RPL39 promoter), integrated at the HO locus, was introduced into the GFP collection to mark the cell in order to facilitate automated image analysis. Micrographs were acquired using a confocal microscope (Opera, PerkinElmer). Eight micrographs were imaged (at 1331×1017, 12 bit resolution) from each strain, 4 in the red channel and 4 in the green channel, yielding a dataset of 44 Gb of image data.

### Image correction

It was noted that the background noise had a mean and variance that was not uniform across the image. Therefore, we defined a background image that was subtracted from each image. This background image was obtained by averaging all the images. The background image intensity accounts at most for a third of the RFP signal expected in mother cells, except for several defective CCD pixels which systematically report the same value.

### Image segmentation

For each image, we modeled the background and foreground (cell) RFP intensity levels with Normal distributions. In order to account for punctuate noise, we used a Pseudo-2D hidden Markov model (P2DHMM) [54] to model the dependence of neighboring pixels. In order to recover the maximum likelihood parameters for the Normal distributions and state transition probabilities efficiently, we performed expectation maximization (EM) on both the image under the assumption that image rows are independent, and on the same image where columns are now assumed to be independent. Finally, we infer the probability for each pixel to belong to the foreground, as the average of the two probabilities that were calculated when we assumed rows and columns were independent.

### Edge Distance map

Given an image for which we know the probability of each pixel to be from the background, we want to define a map of geometric distance to background for each foreground pixel. We estimate this quantity using an iterative motion on the image grid (which includes diagonals and knight moves), where transitions from a point deterministically select the neighbor through which the shortest path to background is expected. We then compute the expected path length under the assumption that pixels reached along paths have background/foreground state transitions described by a HMM with the parameters inferred from the segmentation. The transition probabilities for diagonal and knight moves are obtained by exponentiation of the transition matrix by the distance between the two points. Since it is enforced that transitions are only allowed from point of higher expected distance to lower ones, distances can be computed directly by dynamic programming, in linear time of the number of pixels in the image. The Edge Distance map (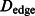) has several uses in our pipeline: to generate the clump contours, as a quality measure for identified objects and to evaluate the distance of a protein to the periphery.

### Robust regression for ellipses

We used robust regression for matching ellipsoidal shapes to the contour of the segmented area. An ellipse is characterized to be the set of points for which the algebraic error 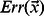 [55] is zero:
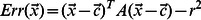
(5)where 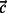 is the coordinate of the ellipse centre and r is an additional parameter, proportional to the radius of a circle for a fixed matrix A.

The matrix A may make the set of points with zero algebraic error correspond to a hyperbole or a line, and a superfluous scale parameter is observed in this parameterization. We therefore constrain the form the of matrix A:
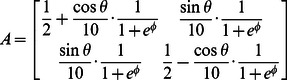
(6)where 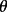 is the angle corresponding to the orientation of the major axis, and 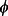 is a parameter which determines the eccentricity of the ellipse. This choice of this matrix to ensure that for any value for the set of 5 parameters (in equation 5) generates an ellipse with minor to major axis length ratio larger than 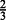, as they are both determined by the eigen values of the matrix A and then scale with the parameter ‘r’ [55].

Contour pixels are first identified by finding foreground pixels which are 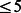 pixels away from some background pixel (using the Edge Distance Map described above). Initial guesses for ellipses are generated by first fitting a circle to 3 randomly sampled contour points (that circle is unique). Initial guesses are rejected if the circle does not fit within the rectangle clamping the contour points, or if the center is a background pixel. The initial guess ellipse will be set to match width (diameter) and center of an accepted circle. A small eccentricity corresponding to 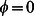 and a random angle 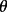 (drawn uniformly from 0 to 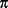) is used to define its remaining parameters.

If the set of contour pixels matches a single ellipse, we could directly update the ellipse coordinates by minimizing the sum of the algebraic error of all contour pixels. However, if the set of contour pixels is best explained by several ellipses, the sum of algebraic errors is likely to have local minima that are not close to any of the true ellipse parameters. Therefore, we use robust regression [56] and minimize the objective function:
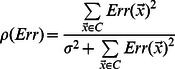
(7)where C is the collection of coordinates of contour pixels and 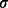 is the expected error, which is chosen to be 5, matching the thickness of the contour. This effectively weights down the importance of contour points with large deviations to the current ellipse, so that the many local minima can correspond to actual ellipses.

Upon convergence, we discard ellipses that are not bounded by the clamping rectangle, or that have a background pixel at the center. Since a large number of local minima are expected, we generate about 10 fold more sets of ellipse parameters than the number of expected ellipses (based on number of contour pixels) and select the ellipse with the best fit. Once we have identified the best ellipse, we remove all contour pixels that have an error smaller than 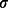, and find the next ellipse using the remaining contour pixels using the same procedure. Since some missed lone pixels may remain, we reject the ellipse and remove the corresponding pixels if the ellipse width is less than 3 pixels or if the number of removed contour pixels accounts for less than 10% of the amount expected from the ellipse parameters and known contour width. This process is repeated iteratively, until no more contour pixels can be removed. The running time of the segmentation is linear in the number of pixels in images, and the running time of cell-finding is linear with the number of randomly sampled circles for the initialization of geometric ellipse fit. On a single 2.83 GHz Intel core, 98 seconds were required to analyze a single 1331×1017 image, which on average contained 82 cells and 31 artifacts.

### Cell shape

We want to precisely recover the cell shape, as we will be considering the size of the bud object as a cell-stage indicator, and the position of the bud neck as a point of interest for uncovering cell-stage dependent changes of protein localization. Because cells are not exactly ellipsoidal in our images, we sought to capture foreground pixels which partition the cell clumps into non-overlapping cell areas (which we refer to as ‘shapes’). In our images, cells are often separated by dim pixels, so we force boundaries to match these dim areas.

We first use the watershed [57] transform to identify regions of the clump that are entirely contained within single cells. For each pixel which brighter than any of its neighboring pixels, we find the set of pixels (catchment basin) which can be reached by a path of monotonically decreasing intensities [57]. Secondly, we assign each basin to a cell, by finding the ellipse closest to each maximum intensity pixel. The proximity of a point to an ellipse is evaluated using the algebraic error (Equation 5). This procedure ensures that if two neighboring basins are assigned to different cells, we are guaranteed that the boundary pixels are all dimmer than the nearby inner pixels found inside one of the two basins.

Each such basin is then assigned to the closest ellipse, such that the union of these regions forms the ‘shape’. The resulting shapes may be highly non-ellipsoidal (Figure 1iii); for example, if a cell has not be properly fitted by an ellipse, a portion of its area may appended to the area of a neighbor cell instead.

### Cell confidence

In addition to the mean RFP intensity in the object, we define three shape measurements based on geometrical properties of ellipses and circles. First, we compute the best fit of an ellipse to an arbitrary shape ‘
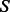
’ by evaluating the following 6 statistics on the coordinates of pixels in the shape (eq. 8).
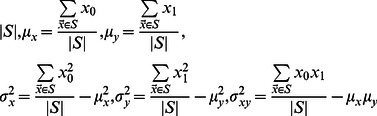
(8)where 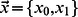 is the coordinate for a pixel from the shape ‘
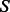
’, and 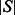 is the number of pixels in the shape (cell size). A function defined on 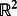 which takes the value ‘D’ within the area of an arbitrary ellipse has 6 degrees of freedom as well:
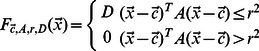
(9)We can derive that there is a closed form for the parameters of the above function that makes the corresponding statistics defined on a continuous space match the statistics from the pixel coordinate of any shape. For instance, the center of the fitted ellipse will correspond to the center of mass of the provided shape:
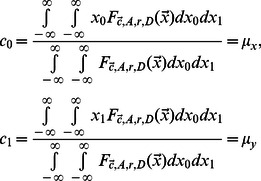
(10)The major and minor axes length (‘
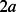
’ and ‘
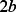
’) are the square root of the two solutions to a quadratic equation:
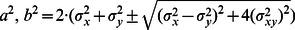
(11)Finally, the recovered density ‘
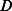
’ is the ratio of number of pixels to fitted ellipse area. Since the coordinates are drawn from a bitmap, we observe that the measured densities typically are bounded above by 1, except for the smallest objects. Any shape whose density is above or equal to 1 is assigned to the artifact class, otherwise we use the following first quality measure:
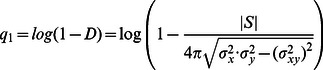
(12)The second quality measure is based on the relationship between the perimeter and the area of an ellipse. We compute the perimeter of the shape by counting the number of pixels that have 3 or more background pixels among their 8 neighboring pixels. The theoretical relationship between the perimeter length of an ellipse and its parameters has no simple form, but may be approximated using the Ramanujan first approximation [58]:
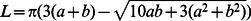
(13)The log ratio for the number of contour pixel to Ramanujan ellipse perimeter length approximation is our second confidence measure 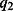.

A third quality measure captures the deviation of the shape to a circle, by reporting the log coefficient of variation of the sum of the distance to the ellipse center and the distance to the edge for each pixel in the area(eq 14). In a theoretical circle, there should not be any variance, since the two quantities are to sum up to be exactly the radius of the circle.
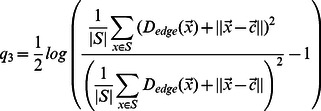
(14)The last quality measure is the mean RFP intensity 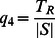. We model each of the quality measures using a Normal distribution. We observe that the quality measure spread displays a non-trivial dependency on cell size. For this reason, we define 7 Normal distributions, for each of the 4 quality measures, which correspond to the distribution of quality measure for 7 bins of cell sizes. The quality measure vector is then modeled by the linear interpolation of a pair characterized random variables 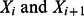
(eq. 15).
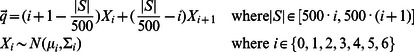
(15)where 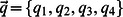 and the 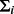 are diagonal covariance matrices.

We used the automatically identified shapes that were mapped to the 4305 manually identified cells in order to infer the parameters of the normal distribution at the 7 sizes (7 means and 7 standard deviations). In order to define the posterior probability of cell, it remains to characterize the uniform distribution for the non-cell objects and the mixing parameter 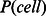 (eq. 1). The uniform distributions were chosen to correspond to the extremum in quality measure obtained from the complete collection of identified objects that have not been labeled as artifacts. Finally, we used soft expected maximization (soft-EM) [59] on the complete collection to infer the mixing parameter, which rapidly converged to 9.9% as all other parameters are already predefined.

### Evaluation of cell identification performance

In order to evaluate the accuracy of our cell identification method, we first compared the automatically identified ellipses to a set of 4305 ellipses that had been drawn around cells manually. We assigned each manually identified ellipse to the automatically identified ellipse with closest center. We found that for 94.2% of manually identified ellipses, there is an automatically identified one with center occurring within 10 pixels. In these cases, the average distance between the centers was 1.86 pixels (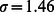). The correlation between the areas of the automatically identified and manually identified matched ellipse pairs was 0.882.

We next compared the center and area of the automatically identified ‘shapes’ to the set of manually drawn ellipses. Here, 92.3% of the manually drawn ellipses have a corresponding recovered shape that has a center within 10 pixels of the manually drawn ellipse center. For these, the mean distance between the shape and the manually drawn ellipse center was 1.41 pixels (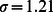). The area of the ‘shapes’ have a correlation of 0.953 with area of the automatically identified ellipses, and 0.928 with the area of manually drawn ellipses. Hence, identifying the ‘shapes’ (the hybrid operation of assigning the watershed regions to their closest ellipse) produces cells that are on average closer both in location (1.41 vs. 1.86 pixels) and size (correlation 0.928 vs. 0.882) to the manually drawn ellipses than the automatically identified ellipses. We note that the ‘shape’ -based analysis led to a slight reduction in the fraction of cells identified (92.3% from 94.2%) but this was acceptable to us in the context of the improvement in cell size estimation (0.928 vs. 0.882 correlation) because we use the cell size as an indicator of cell stage.

In order to compare the accuracy of the simple cell-finding method described above with an established method for cell identification, we compared our results to Cell profiler [60]. For background correction, we used the polynomial fit to the ensemble of images, and subtracted the resulting amount from each image. We identified the primary objects under Otsu global threshold method, and used the ‘Shape’ method for defining boundaries between objects and to distinguish the clumped objects. We chose this method because the Cell profiler documentation suggests it as proper to recover round objects in clumps. Using the same method described above for our pipeline, we compared the cells identified by Cell profiler to the manually drawn ellipses. We found that 89.0% of the manually drawn ellipses have a corresponding identified cell within 10 pixels of their area centre. The mean distance in the paired centers was 2.23 (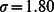) and the correlation in object sizes 0.876. Although these statistics are slightly lower than for our simple methods, it did perform significantly faster, identifying the cells in a typical image in 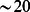 seconds.

In addition, 139 artifacts were manually identified. We used this set to compute the false positive rate by pairing automatically identified cell areas to the manually identified cells and artifacts (Suppl. Figure S2). We also computed the false-positive rate as a function of cell probability threshold. For example, filtering all cells that have a cell probability below 0.8 reduces the false positive rate. This is in agreement with previously reported results using post processing [61]. Since we we found that the number of cells is critical for the robustness of the time profile estimates (Suppl. Figure S3) and that small buds have systematically lower cell probability estimates (Suppl. Figure S1B), we prefered not to choose a hard threshold. Indeed, we found that using a 0.8 cell probability threshold reduces the robustness of the time profiles (Suppl. Figure S3B). We also found that applying this threshold would discard 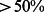 of the small buds which were used to define the first four of our ten cell-stage time points.

### Protein expression measurements

We characterize the protein expression phenotype within each cell object using the absolute intensity of the GFP, as well as geometrical distances between proteins to identified points of interest. In both cases, we use the RFP signal to normalize the observations made for the GFP signal. The RFP intensity was found to be dependent on the object size, so we characterized the expected RFP, 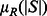, and used to normalize the GFP signal by the fold difference to the expectation of the mean RFP intensity (eq. 2). We defined 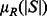 using three linear function segments which fits the mean level of RFP in the 1.4M automatically identified cells:
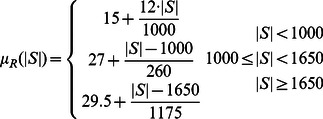
(16)Some of the morphological distances require us to identify the coordinates of a point of interest; the cell center, protein mass-center and bud neck position are obtained by averaging the coordinates of the cell pixels, of GFP-tagged proteins and Mother-bud separation contour pixels, respectively. Assuming GFP intensities are proportional to protein amount, we derive the expected value for geometrical distances with respect to the position of a randomly selected protein. The position of cell center, protein mass center and bud neck are given by:
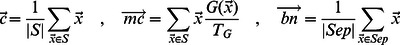
(17)where 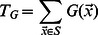 is the sum of GFP intensities and ‘
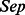
’ is the set of contour pixels which separates the bud from the mother cell. The other 2 distances have a slightly different form: first, the distance to the perimeter for any coordinate has been computed using Edge Map distance, so that:
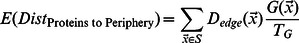
(18)Second, we derive the equation for the expected distance between proteins:
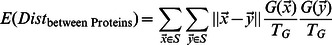
(19)Once again we use the RFP marker to normalize these distances. In the case of distance between proteins, the distance is normalized by the expected distance between a protein and a RFP marker. For that case, the reported log ratio representing a morphological distance would be:
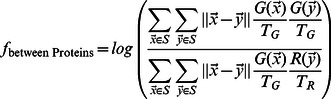
(20)


### Time profiles

First, we model cell stage as a function of the bud size. Under the assumption that the bud volume increases at a constant rate, we expect that time scales linearly with 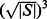. Because we have a number of identified cells and distribution of object size that varies throughout the collection of 4004 yeast strains, a common basis is required to enable comparisons between the expression of different proteins. For each strain, time series are defined as expected feature values for objects observed at 10 equidistant cell stage keypoints 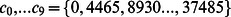. We use local regression (LOESS) to infer the mean and variance at each keypoint (eq. 21), where the ‘
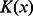
’ is Gaussian kernel function with bandwidth parameter equal to 1700. In addition, because we have developed a probabilistic cell confidence, which assigns to each identified cell a posterior probability of being a properly identified cell, we use the cell confidence to compute a weighted average, which is the expected profile conditioned on each identified object being drawn from the cell class:
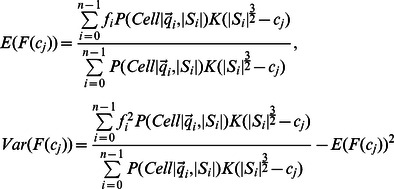
(21)where 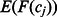 is feature value that is expected at the cell stage keypoint 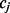 from feature values 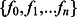, which are measured for the 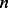 identified object. 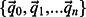 are the quality measures for each shape and 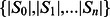 are cell sizes for bud objects.

### Maximum likelihood agglomerative hierarchical clustering

Each protein profile is a vector of means and variances of observations. We use the Maximum likelihood clustering criterion [37] (eq. 22) in order to agglorameratively join pairs of protein profiles, proteins to cluster profiles, or pairs of cluster profiles:
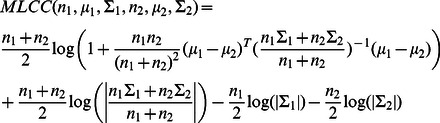
(22)where 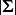 is the determinant of a covariance matrix. This criterion is the log likelihood ratio for two cluster of size 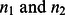 to have their protein profiles modeled as two multivariate Normal distributions (with their corresponding parameters 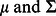), to a single multivariate Normal model explaining both expression groups.

Initial cluster profiles are build from individual protein profile, which corresponds to 12 concatenated time series of feature values. As such, initial covariance matrices are diagonal matrices whose values were estimated from the LOESS(see 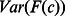 in eq. 21). New cluster profiles are characterized by a multivariate normal distribution whose parameters are obtained from merging two previous cluster profiles (eq. 23).
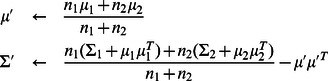
(23)where ‘
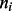
’ are cluster sizes and 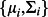 are normal distribution parameters for merged cluster profiles.

### Class profiles

The 4004 proteins were grouped based on exact correspondence of subcellular location annotation, as defined by Huh et al. [14] (Suppl. Table S1). 22 classes correspond to unique subcellular locations. We merged member profiles into a class profile (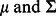) using the operation defined above (eq. 23). The Bhattacharyya metric (eq. 24) was used to compare each class profile (Suppl. Table S1) and class profiles were clustered using euclidean distance (Suppl. Figure S5A).
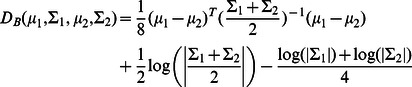
(24)


### Enrichment of functional annotations

Protein subcellular location was characterized by Huh et al. [14] by assigning one or many annotations to each protein. We report about the enrichment of either separate annotations and/or exact localization set correspondence. For example, proteins that were only annotated to be nuclear obtained the label ‘pure nucleus’ and new labels, such as ‘nucleus AND cytoplasm’ were reported. The GO and PFAM annotation were obtained from Uniprot/SwissProt [51]. In the reported hierarchical clustering results (Figure 6), clusters were manually selected and the hypergeometric distribution was used to model the occurrence of annotations of proteins within them. Bonferroni correction was applied to the P-values (1990 hypotheses, accounting for 3.3 in log scale).

### Significance of hierarchical clustering

To assess the significance of the hierarchical clustering, we performed permuation tests. For each protein annotation, we find the cluster that yields the smallest P-value for annotation enrichment. We then assess the statistical significance of the sum of the smallest log P-values, ‘S’, by defining two background distributions for ‘S’. In the first, we preserved the structure of the tree, but chose random proteins to assign to each leaf. In the second, we preserved the structure of the tree, but randomly replaced the proteins with other proteins that had exactly the same set of annotations of subcellular localization. In other words, for this second ‘localization constrained’ permutation, we only allow proteins of identical characterization in subcellular location terms to be permuted, so that any enrichments of subcellular location (as displayed in Figure 6) will be preserved for any permutation. We found that the statistic ‘S’ was systematically higher in the 10000 permutations than for original hierarchical cluster. Therefore, we report the corresponding Z-score, but we note that background distribution for ‘S’ is not necessarily a normal distribution (Suppl. Table S3).

In order to evaluate the resolution of functional enrichments in the hierarchical clusters, we computed the significance for subsets of annotations. We show in supplementary table S3 that complexes characterized by GO annotation are found significantly enriched, and that the ribosomal and proteasomal proteins, which typically show high protein abundance, have a limited contribution in the sum. In addition, we applied the statistical tests on 14 subsets of GO annotations based on the number of annotated proteins. This analysis was also performed on 5 alternative hierarchical clustering results: This allows us to evaluate the robustness of the results to a change of clustering algorithm (Maximum likelihood clustering, Euclidean metric with complete linkage, Correlation metric with complete linkage), and the usage of the cell confidence (as a weight or using 0.8 as a filtering threshold) (Suppl. Table S2, S3, S4, S5).

### Data and code availability

Hierarchical clusters are available to be browsed online at: http://www.moseslab.csb.utoronto.ca/louis-f/unsupervised/. In addition, the source code for the cell identification and feature measurements, the set of 17 images in which 4305 ellipses corresponding to cells and 139 ellipses corresponding to artifacts were manually drawn, as well as a table of feature measurements for all 400 K mother-buds pairs are available.

## Supporting Information

Figure S1 Confidence estimates for automatically identified cells a) Histogram of Cell probability for Automatically Identified Objects. Cell probability is calculated for each of the 1.3 million identified cells as defined in the text. The assigned cell probabilities are displayed using 100 bins. The majority of the identified shapes have a probability to belong to the cell class which is above 95%. b) Dependence on bud size for cell confidence on bud cells. The set of 405359 identified buds was partitioned into 10 groups based on bud size, such that each group had the same number of cells. The mean and standard deviation in the measured cell probabilities is shown (grey bars). Smaller buds tend to have lower cell probabilities.
(TIFF)

Figure S2 ROC curve for cell identification with confidence scores. A test set of 4305 cells and 139 challenging artifacts were identified by manually drawing ellipses around objects in images. Automatically identified cell areas were paired to manually drawn ellipses if they were within 10 pixels. Other manually identified cells were considered false negatives. The false-positive rate (number of artifacts/number of predictions) and true positive rate (or recall, which is the number correctly identified cells/number of manually identified cells) are plotted as a function of cell confidence. As a reference, we also display the performance using a Cell profiler pipeline (red diamond, see ‘Evaluation of cell identification performance’ in methods) and the baseline accuracy of our method (blue triangle) without a cell probability cutoff. The expected performance of random guessing corresponds to y = x in this plot (thick black trace).
(TIFF)

Figure S3Global evaluation of the robustness of time profiles - a) We used the Jackknife [30] estimate of sampling variability observed in time profiles computed from local regression (LOESS [29], eq. 21). The measured variances were normalized by the total cell-to-cell variance in the corresponding feature, so the robustness of all the 4004×10×6×2 time points are presented. The number of mother-bud pairs identified, which varies from protein to protein, affects the robustness of the estimates. Bars represent fraction of the total variance due sampling for proteins with 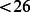 mother-bud pairs (red bars), proteins with 26–99 mother-bud pairs (green bars) or proteins with 100–307 mother bud pairs (blue bars). b) To evaluate the effect of our cell probability weighting, we computed the time series for cell data without weighting by cell confidence. Instead, any cell that had a cell probability below 0.8 was ignored from the analysis. Hence, all Mother-Bud pairs that have high enough confidence for both objects equally contribute to the time-profile estimation. The jackknife estimate reports slightly higher levels of sampling variability overall using the hard threshold.(TIFF)

Figure S4 Evaluation of significance of cell-stage deviations in protein expression. We display the local regression time profile for the intensity of the proteins Ash1, Cdc6 and Sic6 (blue traces and symbols). The background distribution of intensity estimated at each time point is produced by permuting the cell-stage estimates for each identified mother-bud pair 10000 times (red traces and symbols. Error bars represent the standard deviation of the empirical distribution of the permutations). Numbers below the time points display P-values for the deviation of the time point from the real data (positive and negative deviations in the 2.5% tails of the empirical distribution of the permutations are reported).
(TIFF)

Figure S5Comparison of time profiles for different subcellular locations a) Hierarchical Clustering of the class profiles based on Euclidean distance. Colours of location names indicate the 4 groups of subcellular locations that were defined based on biological relationships. b) Average Bhattacharyya distance between subcellular location class profiles within biologically related groups (between members, blue bars) is smaller than the average distances between these class profiles and those that are not biologically related (to non-members, red bars). We note that the sum of the difference in mean distance (difference between blue and red bars) is significantly lower than expected by chance (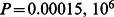 permutations of the subcellular locations that belong to each biological group).(TIFF)

Figure S6 a) Mitochondrial proteins show punctae in buds. We expected single punctae to arise in small buds for mitochondrial proteins based on the time profiles of our simple features. Visual inspection of the cell populations of 5 randomly chosen mitochondrial proteins allows us to identify mother-bud pair examples that appeared to correspond to our expectation (punctae indicated with arrows). For comparison we include mother-bud pairs with smaller or larger buds (top and bottom rows, respectively). Neither of these groups shows the single bright spot of protein expression. Images have been contrast enhanced to enable visualization of dim cells. b) Visualization of hierarchical clusters obtained using alternative parameters. On the left, the hierarchical clustering was performed on time profiles that used a cell confidence threshold (of 0.8). On the right, the correlation metric and complete linkage hierarchical clustering was used. The inclusion of the nucleus in the bud is indicated with the dotted yellow line, and the characteristic time for proteins to reach their maximum distance to the bud neck is shown in light blue braces for Mitochondrion, and light orange brace for ER.
(TIFF)

Figure S7 Examples of proteins in our dynamic bud cluster. Images are representative of patterns for each protein. The contrast of each image has been enhanced to display patterns more clearly. These proteins were not previously annotated as showing bud-related patterns by Huh et al. [14] or Chen et al. [16]. The top 6 proteins (indicated using a green bar) are found localized to the bud tip and/or bud neck, so that they exhibit a dynamic bud pattern. For Tpo3 (indicated using a red bar), it is doubtful whether this is the case: Tpo3 typically appears in the cell periphery and nuclear periphery. Hence, Tpo3 is an example of a negative prediction of a dynamic bud protein.
(TIFF)

Table S1 Distance between subcellular localization class profiles. For each of the 22 subcellular location defined by Huh et al. [14], we defined the average expression profile for each of the protein that was annotated as appearing in only one localization class. The maximum likelihood clustering agglomerative method was used to define multivariate normal distributions representing a ‘profile’ for each class (see ‘Class profiles’ in Methods). The Bhattacharyya metric (eq 24) was used to evaluate the distances between profiles, as it is not dependent on the number of proteins ‘n’ that defined each profile, as opposed to the maximum likelihood criteria(eq 22).
(XLSX)

Table S2 Enrichment of subcellular localization in hierarchical clustering results. For each subcellular location, the inner cluster that shows the most significant enrichment was identified within the hierarchical clusters. For 6 hierarchical clustering results, a P-value is reported for the significance of the enrichment of each localization class. The enrichments were computed for ‘pure’ patterns, and ‘partial’ patterns.
(XLSX)

Table S3 Significance of functional annotation enrichment in the hierarchical clustering results. For each annotation, the most significant enrichment among the cluster found within the hierarchical clustering results was evaluated using the Hypergeometric distribution. The sums of the log P-value (Log pvalue sum) were computed for various sets of functional annotations. For example, GO 19–24 is the GO annotations assigned to between 19 and 24 proteins in our set of 4004. Pfam and Complexes indicate the annotations of Pfam domains and protein complexes. The significance of sums of log P-values were evaluated by generating the background distribution of sum of log P-value occurring by permuting the proteins in the hierarchical clusters. To reject that the enrichments are explained by enrichments in subcellular location alone, we constrained the permutation to only protein of identical assessment by Huh et al. [14]. In all cases, 10000 permutations never generated values for the ‘S’ statistics that were as extreme as observed in the original hierarchical clustering results. For that reason, significance is also reported as a Z-score although the background distribution is not necessarily a Normal distribution. Using a different metric or defining time-profiles using only cells with confidence score above a threshold cells produces similar observations.
(XLSX)

Table S4 GO annotations for protein complexes with highest enrichment in hierarchical clustering results. The cluster with most significant annotation enrichment is found for each annotation. We correct for multiple hypothesis tests (277 complexes) using the Bonferroni correction, which accounts for 2.44 in log scale. Enrichments within 5 other hierarchical clustering results are also reported. For instance, the all four MCM subunits are clustered in 5 out of the 6.
(XLSX)

Table S5 Pfam annotations with strongest enrichment. The cluster with most significant annotation enrichment is found for each annotation. We correct for multiple hypothesis tests (671 Pfam) using the Bonferroni correction, which accounts for 2.88 in log scale. Enrichments within 5 other hierarchical clustering results are also reported.
(XLSX)

Table S6 List of Dynamic Bud Proteins. For each protein from figure 8, the Huh et al. [14] subcellular location, the revised location proposed by Chen et al. [16] and the Cellular compartment from SGD [45] is reported. We note that proteins that are not identified to be bud neck or actin by Huh et al. typically have subcellular location that disagree between annotation sources.
(XLSX)
