# Supplementary figures and images for: Unsupervised Clustering of Subcellular Protein Expression Patterns in High-Throughput Microscopy Images Reveals Protein Complexes and Functional Relationships between Proteins
Source: PLoS Comput Biol. 2013 Jun 13;9(6):e1003085. doi: 10.1371/journal.pcbi.1003085 (PMC3681667; doi:10.1371/journal.pcbi.1003085)

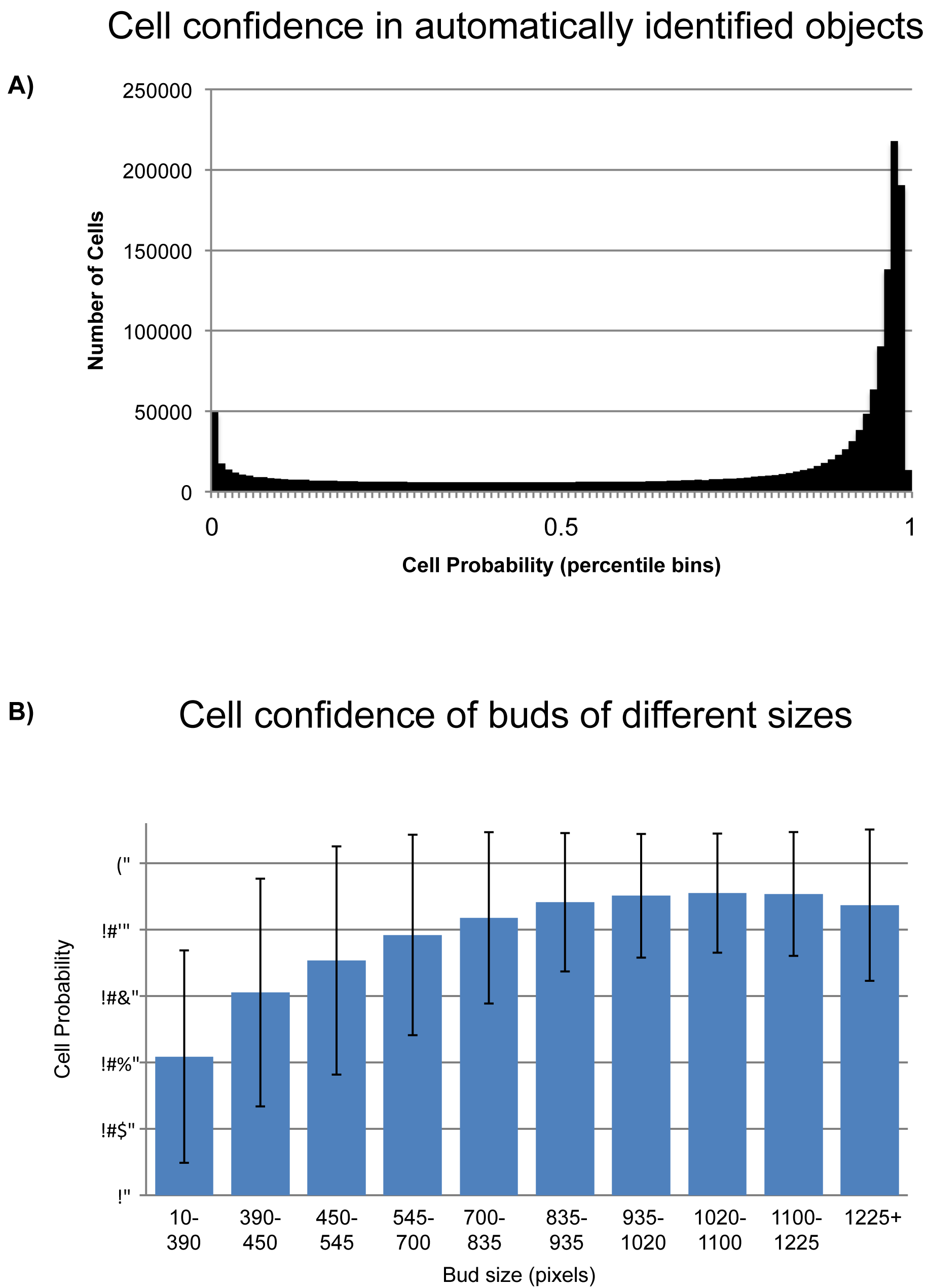

Supplement: Figure S1 — Confidence estimates for automatically identified cells a) Histogram of Cell probability for Automatically Identified Objects. Cell probability is calculated for each of the 1.3 million identified cells as defined in the text. The assigned cell probabilities are displayed using 100 bins. The majority of the identified shapes have a probability to belong to the cell class which is above 95%. b) Dependence on bud size for cell confidence on bud cells. The set of 405359 identified buds was partitioned into 10 groups based on bud size, such that each group had the same number of cells. The mean and standard deviation in the measured cell probabilities is shown (grey bars). Smaller buds tend to have lower cell probabilities. (TIFF) [file pcbi.1003085.s001.tiff]

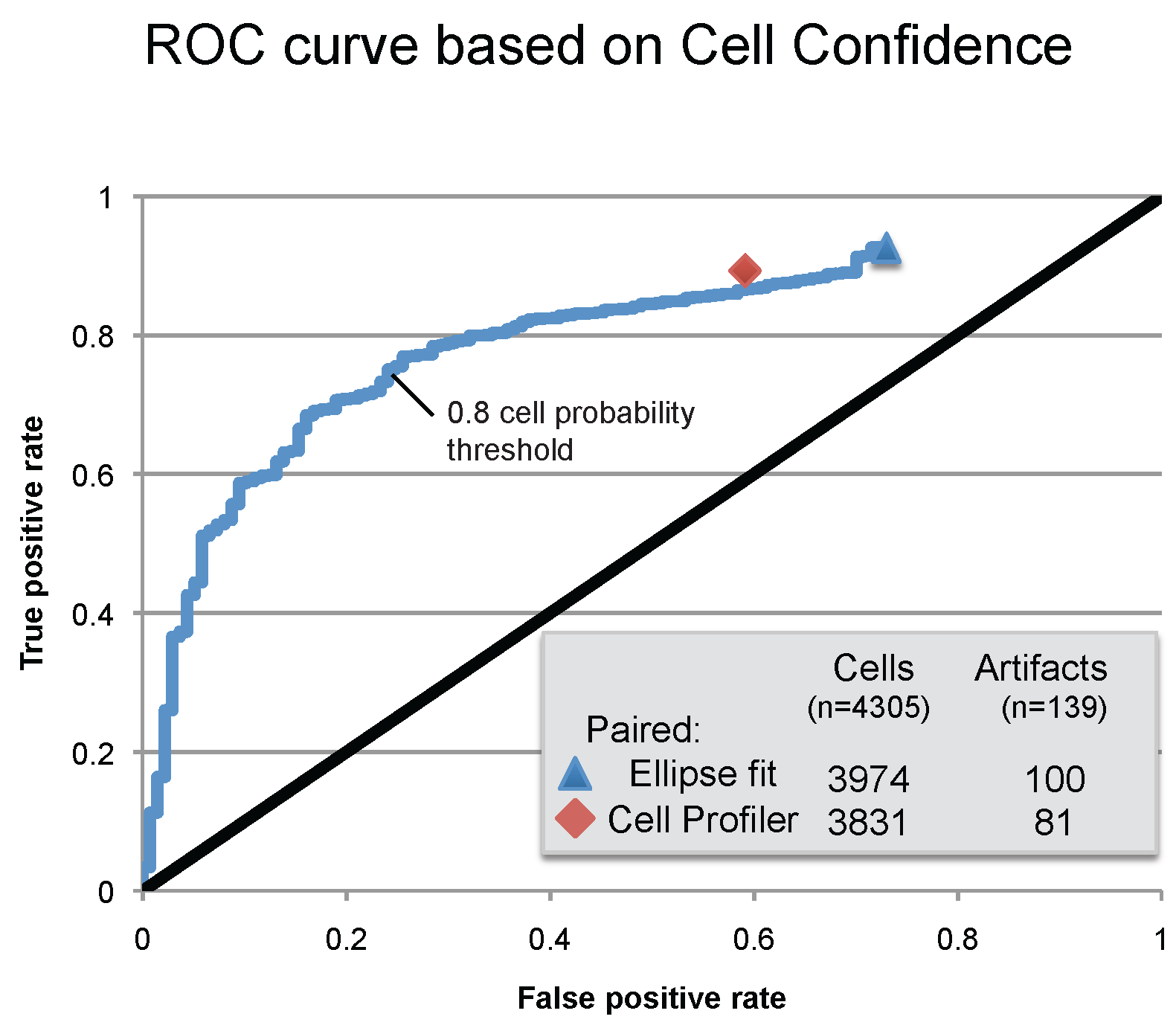

Supplement: Figure S2 — ROC curve for cell identification with confidence scores. A test set of 4305 cells and 139 challenging artifacts were identified by manually drawing ellipses around objects in images. Automatically identified cell areas were paired to manually drawn ellipses if they were within 10 pixels. Other manually identified cells were considered false negatives. The false-positive rate (number of artifacts/number of predictions) and true positive rate (or recall, which is the number correctly identified cells/number of manually identified cells) are plotted as a function of cell confidence. As a reference, we also display the performance using a Cell profiler pipeline (red diamond, see ‘Evaluation of cell identification performance’ in methods) and the baseline accuracy of our method (blue triangle) without a cell probability cutoff. The expected performance of random guessing corresponds to y = x in this plot (thick black trace). (TIFF) [file pcbi.1003085.s002.tiff]

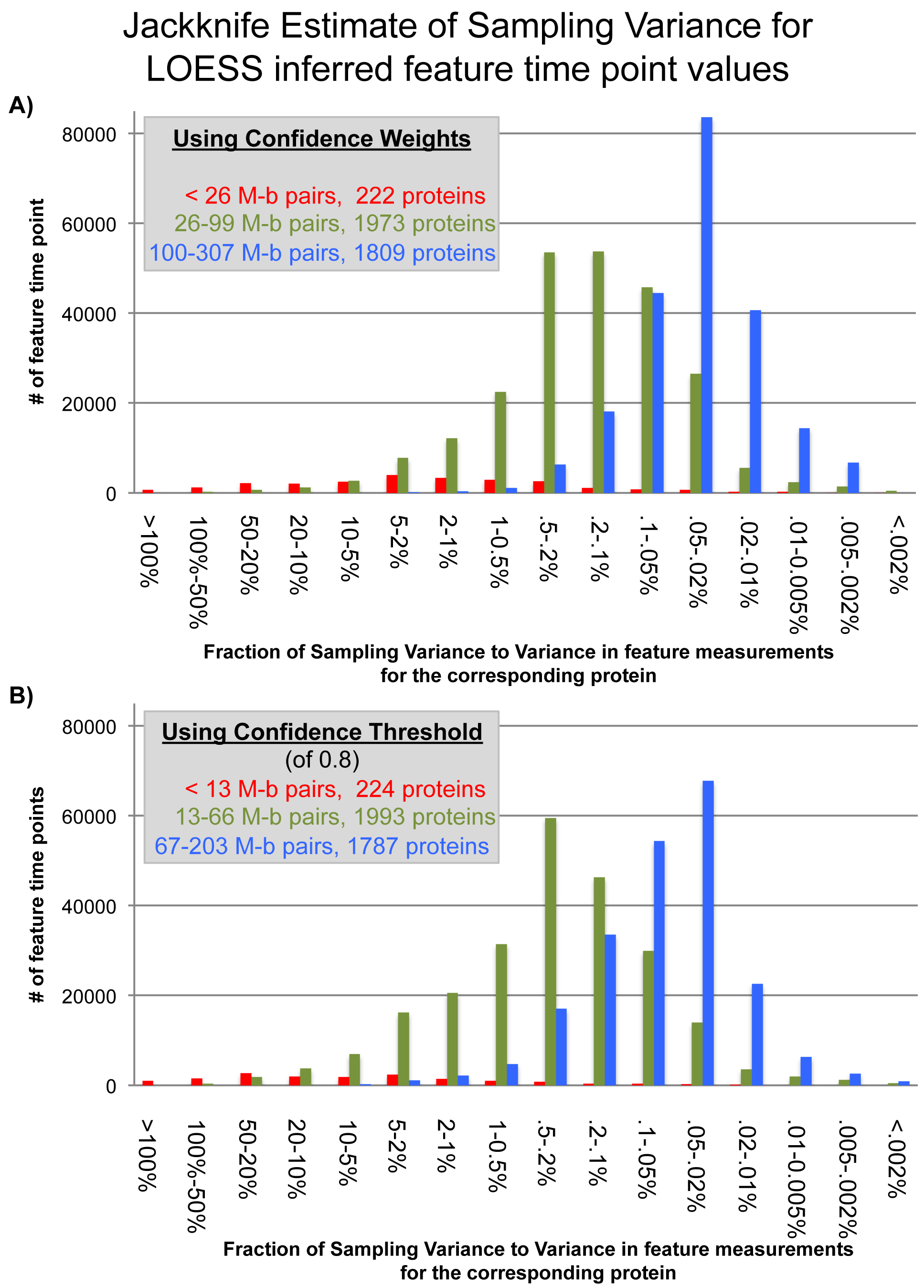

Supplement: Figure S3 — Global evaluation of the robustness of time profiles - a) We used the Jackknife [30] estimate of sampling variability observed in time profiles computed from local regression (LOESS [29], eq. 21). The measured variances were normalized by the total cell-to-cell variance in the corresponding feature, so the robustness of all the 4004×10×6×2 time points are presented. The number of mother-bud pairs identified, which varies from protein to protein, affects the robustness of the estimates. Bars represent fraction of the total variance due sampling for proteins with mother-bud pairs (red bars), proteins with 26–99 mother-bud pairs (green bars) or proteins with 100–307 mother bud pairs (blue bars). b) To evaluate the effect of our cell probability weighting, we computed the time series for cell data without weighting by cell confidence. Instead, any cell that had a cell probability below 0.8 was ignored from the analysis. Hence, all Mother-Bud pairs that have high enough confidence for both objects equally contribute to the time-profile estimation. The jackknife estimate reports slightly higher levels of sampling variability overall using the hard threshold. (TIFF) [file pcbi.1003085.s003.tiff]

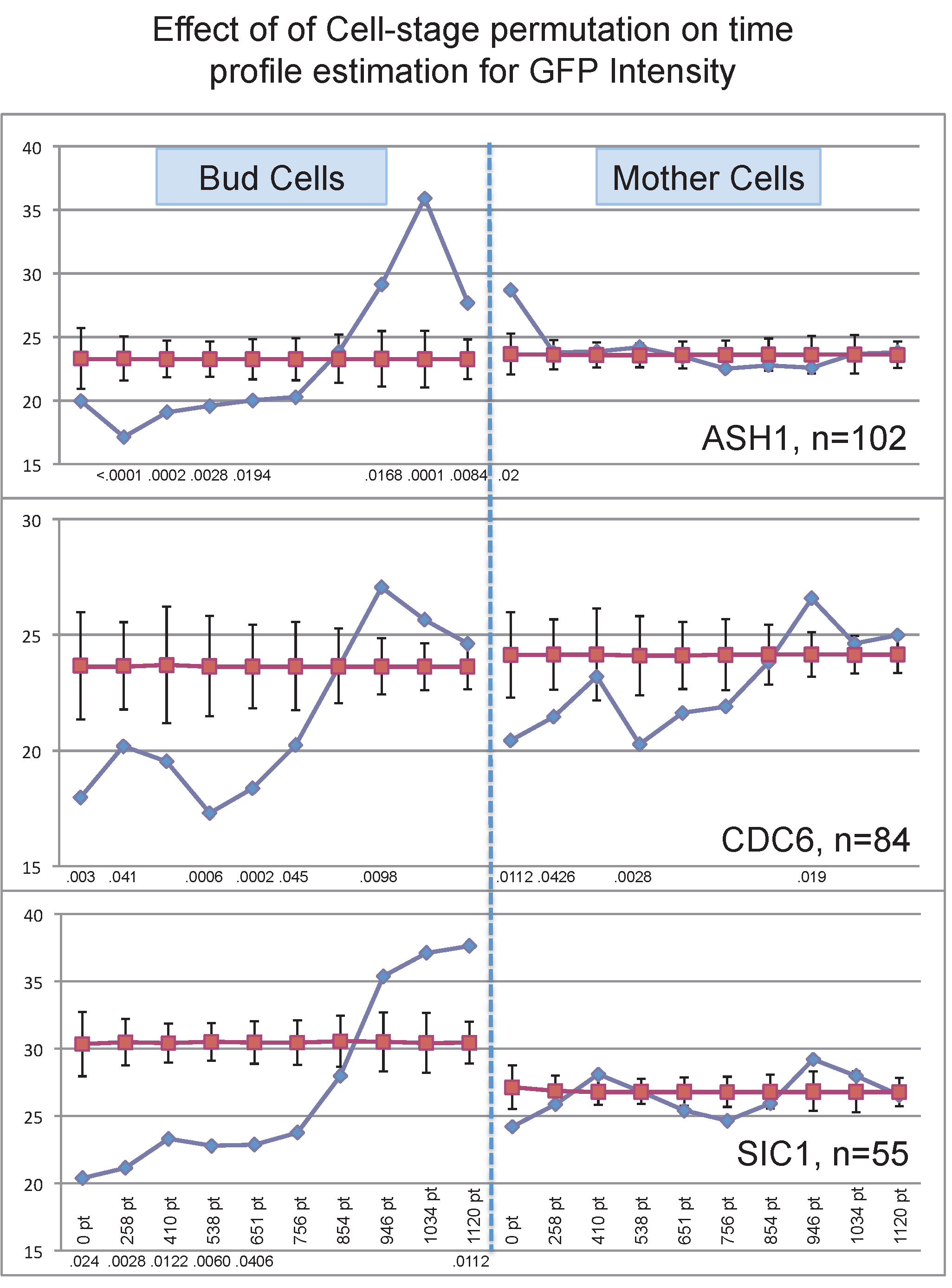

Supplement: Figure S4 — Evaluation of significance of cell-stage deviations in protein expression. We display the local regression time profile for the intensity of the proteins Ash1, Cdc6 and Sic6 (blue traces and symbols). The background distribution of intensity estimated at each time point is produced by permuting the cell-stage estimates for each identified mother-bud pair 10000 times (red traces and symbols. Error bars represent the standard deviation of the empirical distribution of the permutations). Numbers below the time points display P-values for the deviation of the time point from the real data (positive and negative deviations in the 2.5% tails of the empirical distribution of the permutations are reported). (TIFF) [file pcbi.1003085.s004.tiff]

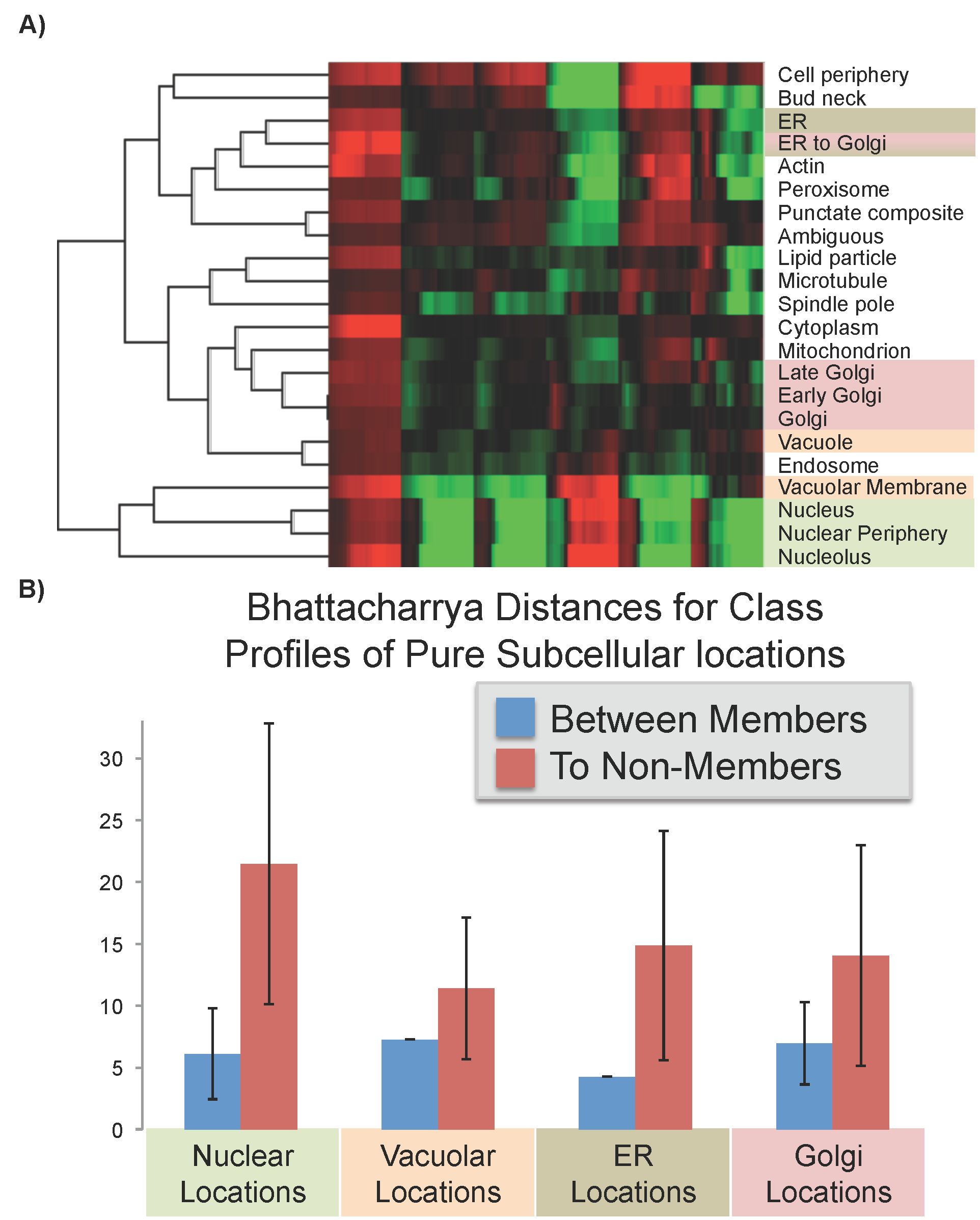

Supplement: Figure S5 — Comparison of time profiles for different subcellular locations a) Hierarchical Clustering of the class profiles based on Euclidean distance. Colours of location names indicate the 4 groups of subcellular locations that were defined based on biological relationships. b) Average Bhattacharyya distance between subcellular location class profiles within biologically related groups (between members, blue bars) is smaller than the average distances between these class profiles and those that are not biologically related (to non-members, red bars). We note that the sum of the difference in mean distance (difference between blue and red bars) is significantly lower than expected by chance ( permutations of the subcellular locations that belong to each biological group). (TIFF) [file pcbi.1003085.s005.tiff]

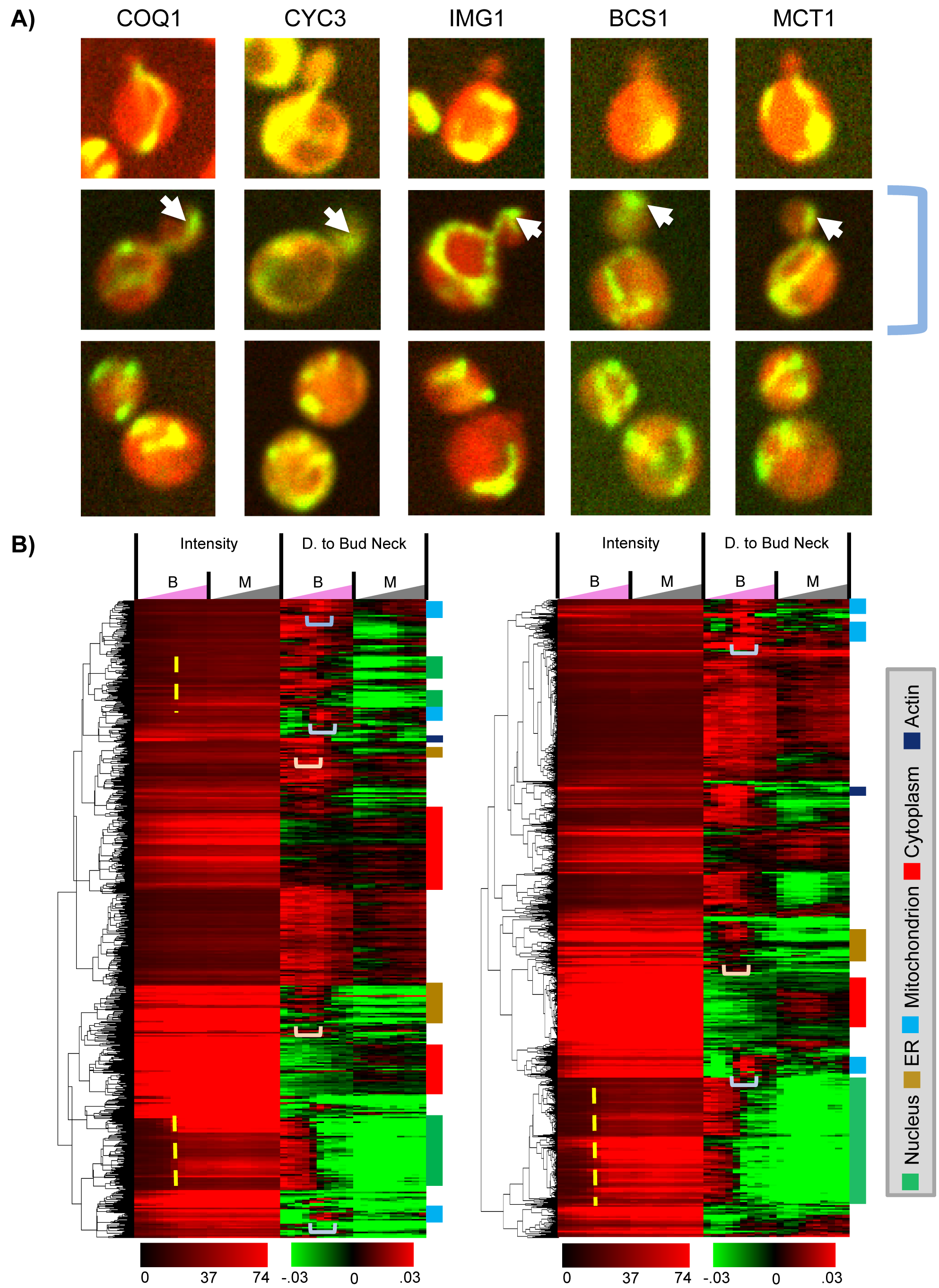

Supplement: Figure S6 — a) Mitochondrial proteins show punctae in buds. We expected single punctae to arise in small buds for mitochondrial proteins based on the time profiles of our simple features. Visual inspection of the cell populations of 5 randomly chosen mitochondrial proteins allows us to identify mother-bud pair examples that appeared to correspond to our expectation (punctae indicated with arrows). For comparison we include mother-bud pairs with smaller or larger buds (top and bottom rows, respectively). Neither of these groups shows the single bright spot of protein expression. Images have been contrast enhanced to enable visualization of dim cells. b) Visualization of hierarchical clusters obtained using alternative parameters. On the left, the hierarchical clustering was performed on time profiles that used a cell confidence threshold (of 0.8). On the right, the correlation metric and complete linkage hierarchical clustering was used. The inclusion of the nucleus in the bud is indicated with the dotted yellow line, and the characteristic time for proteins to reach their maximum distance to the bud neck is shown in light blue braces for Mitochondrion, and light orange brace for ER. (TIFF) [file pcbi.1003085.s006.tiff]

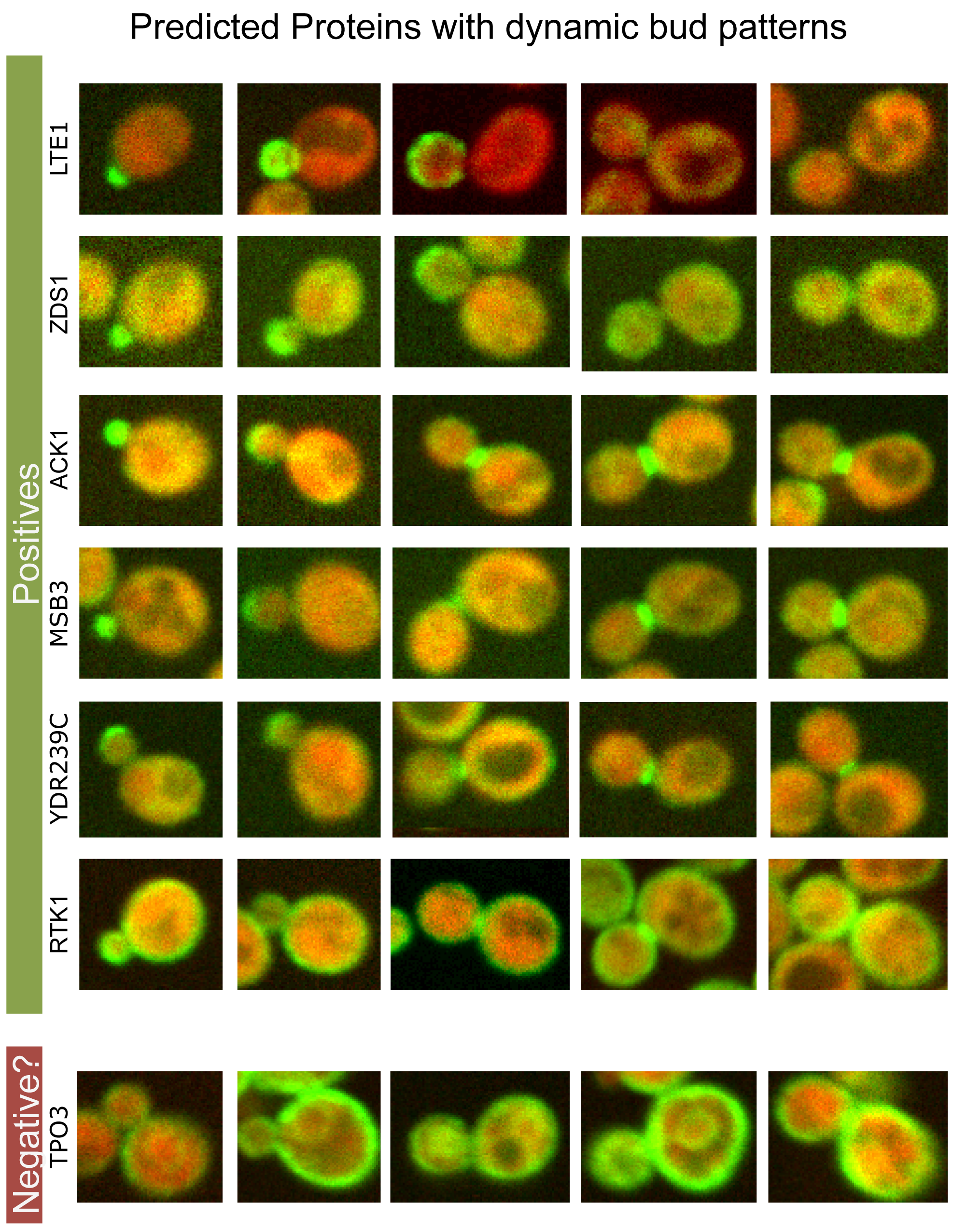

Supplement: Figure S7 — Examples of proteins in our dynamic bud cluster. Images are representative of patterns for each protein. The contrast of each image has been enhanced to display patterns more clearly. These proteins were not previously annotated as showing bud-related patterns by Huh et al. [14] or Chen et al. [16]. The top 6 proteins (indicated using a green bar) are found localized to the bud tip and/or bud neck, so that they exhibit a dynamic bud pattern. For Tpo3 (indicated using a red bar), it is doubtful whether this is the case: Tpo3 typically appears in the cell periphery and nuclear periphery. Hence, Tpo3 is an example of a negative prediction of a dynamic bud protein. (TIFF) [file pcbi.1003085.s007.tiff]
